# Prolintane analogs as hybrid monoamine transporter ligands: Structural determinants and species differences

**DOI:** 10.1016/j.jbc.2025.110903

**Published:** 2025-11-04

**Authors:** Nina Kastner, Mohammad N. Islam, Michael Dybek, Evelyn Roth, Simon Heisinger, Marion Holy, Kathrin Jäntsch, Donna Walther, Thomas Stockner, Michael H. Baumann, Simon D. Brandt, Jason Wallach, Harald H. Sitte, Oliver Kudlacek

**Affiliations:** 1 Center for Physiology and Pharmacology, Institute of Pharmacology, Medical University of Vienna, Vienna, Austria; 2 Department of Pharmaceutical Sciences, Philadelphia College of Pharmacy, Saint Joseph's University, Philadelphia, Pennsylvania, USA; 3 Designer Drug Research Unit, National Institute on Drug Abuse Intramural Research Program, Baltimore, USA; 4 The Alexander Shulgin Research Institute, Lafayette, California, USA; 5 Hourani Center for Applied Scientific Research, Al-Ahliyya Amman University, Amman, Jordan; 6 Center for Addiction Research and Science, Medical University of Vienna, Vienna, Austria

**Keywords:** prolintane analogs, monoamine transporters, structure-activity relationship, serotonin transporter mediated efflux, molecular docking approach

## Abstract

Prolintane is a synthetic stimulant that acts by inhibiting the uptake of dopamine and norepinephrine into neurons. Initially prescribed for attention deficit hyperactivity disorder and narcolepsy, its medical use was discontinued due to concerns about abuse liability. Here, we explored structure-activity relationships for novel fluoro and methyl-ring-substituted prolintanes synthesized *via* a modified one-pot Mannich Barbier reaction. Radiotracer flux assays in transfected human embryonic kidney 293 (HEK293) cells and rat brain synaptosomes revealed that prolintane analogs display potent uptake inhibition at the dopamine transporter (DAT) and norepinephrine transporter (NET), with weaker effects at the serotonin transporter (SERT). Across all compounds, SERT inhibitory potencies were at least 10-fold weaker at human SERT (hSERT) compared to rat SERT (rSERT). Methyl substitution at the 2-, 3-, or 4-ring position enhanced SERT inhibition potency relative to DAT, lowering the DAT/SERT ratio and suggesting reduced abuse liability. Fluorine substitution also enhanced SERT potency relative to DAT, however, to a lesser extent. Interestingly, prolintane and its analogs induced hSERT-mediated ionic currents and [^3^H]serotonin efflux, which was not seen in rat brain synaptosomes. Overall, these findings indicate that prolintane analogs act as potent DAT and NET inhibitors, but they can also act as substrates and evoke serotonin release in cells expressing hSERT, classifying them as hybrid compounds at human monoamine transporters. Our study demonstrates how ring modifications alter prolintane pharmacology, emphasizing the need for future investigations into therapeutic and adverse effects of these compounds. Moreover, the species difference in SERT-releasing activity for prolintane analogs warrants further research.

Prolintane (1-(1-phenylpentan-2-yl)pyrrolidine) (Fig. 1) is an amphetamine-type stimulant (ATS) that was prescribed from the 1950s to the 2000s in South Africa, Australia, and Europe for treating attention-deficit hyperactivity disorder (ADHD)-associated symptoms and narcolepsy (1, 2). Available brand names included Catovit, Promotil, and Villescon as part of a “multivitamin” tablet. The diversion of prescribed prolintane led to its removal from clinical markets, and reports indicate non-medical (*i.e.* recreational) use of the drug and misuse among athletes (2, 3, 4, 5), (https://wellcomecollection.org/works/mfwktq52/items), (https://wellcomecollection.org/works/nm9xpuc6/items).

**Figure 1:**
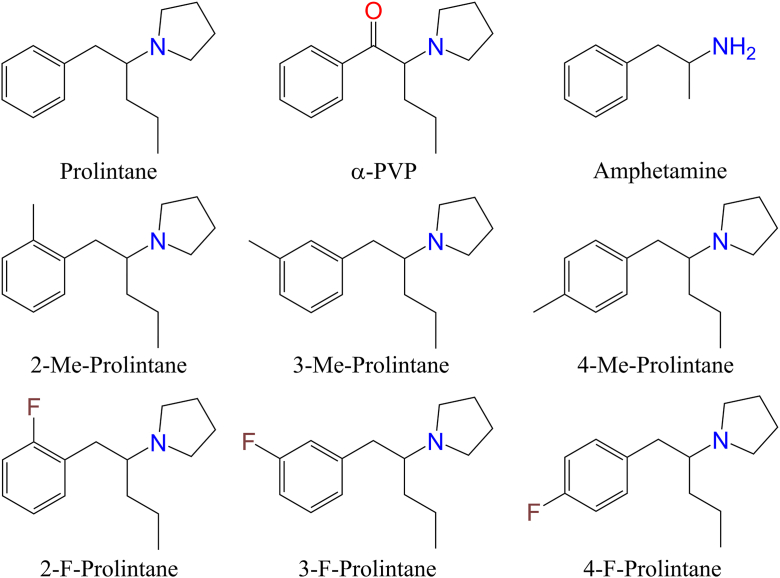
**Chemical structures of prolintane (1-(1-phenylpentan-2-yl)pyrrolidine) and its analogs investigated in this study, as well as structurally related α-pyrrolidinovalerophenone (α-PVP) and amphetamine.** Novel analogs included 2-methylprolintane (1-[1-(2-methylphenyl)pentan-2-yl]pyrrolidine), 3-methylprolintane (1-[1-(3-methylphenyl)pentan-2-yl]pyrrolidine), 4-methylprolintane (1-[1-(4-methylphenyl)pentan-2-yl]pyrrolidine) and 2-fluoroprolintane (1-[1-(2-fluorophenyl)pentan-2-yl]pyrrolidine), 3-fluoroprolintane (1-[1-(3- fluorophenyl)pentan-2-yl]pyrrolidine), and 4-fluoroprolintane (1-[1-(4-fluorophenyl)pentan-2-yl]pyrrolidine) and were prepared as hydrochloride salts. Me, Methyl. F, Fluoro.

ATS enhance activity of the central nervous system (CNS), resulting in heightened alertness, increased energy, and feelings of euphoria (6, 7). Most stimulants elicit their effects by interacting with plasma membrane transporter proteins expressed on monoamine neurons, particularly the solute carrier 6 (SLC6) transporters. The SLC6 family of proteins includes the monoamine transporters for dopamine (DAT), norepinephrine (NET), and serotonin (SERT), which serve to translocate their cognate neurotransmitters from the extracellular space back into the pre-synaptic neuron (8). ATS can interact with transporters as either classic uptake inhibitors similar to cocaine or substrate-type releasers such as amphetamine, which evoke efflux of neurotransmitters *via* reverse transport (9, 10, 11, 12). Prolintane is an uptake inhibitor at DAT, NET, and SERT, but displays much higher potency at DAT and NET when compared to its potency at SERT (13). Thus, prolintane displays a 10-fold higher DAT/SERT ratio when compared to other inhibitors such as cocaine, which suggests prolintane may have greater abuse liability. Consistent with this idea, rewarding and reinforcing effects of prolintane were recently reported in mice after administration of moderate and high doses (2). Like other psychostimulants, the psychoactive effects of prolintane are dose dependent. In humans, the effects of low to moderate doses of prolintane include stimulation, euphoria and insomnia, whereas at higher doses mania and hallucinations can occur (1, 14). In prior clinical studies, prolintane (dose range of 10–30 mg), induced dose-dependent psychostimulant effects, sympathomimetic effects and reduced total rapid eye movement (REM) sleep (15, 16).

Prolintane exhibits structural similarity to the new psychoactive substance (NPS), α-pyrrolidinovalerophenone (α-PVP), a pyrrolidine-containing cathinone that can be regarded as the β-keto analog of prolintane. α-PVP inhibits uptake at DAT and NET, similar to the effects of other pyrrolidine-containing cathinones (13). α-PVP and many of its analogs have been studied extensively in terms of pharmacology and toxicology over the past decade (13, 17, 18, 19, 20, 21) due to their emergence as drugs of abuse (22, 23), (https://www.euda.europa.eu/publications/eu-drug-markets/new-psychoactive-substances_en), but detailed information about their prolintane counterparts is lacking. The widespread availability of cathinone NPS has caused significant concerns to public health, which requires coordinated efforts between health care providers, policymakers, and professionals working in the NPS field (24). At the same time, psychostimulants and related monoaminergic modulators have established therapeutic utility in treating depression, pain, obesity, narcolepsy, ADHD and stimulant-use disorders (25, 26, 27); the rigorous assessment of new stimulant pharmacotherapies is important due to ongoing concerns regarding the potential for abuse liability associated with existing medicines. The current medical treatment options for ADHD include amphetamine, which carries a substantial risk of abuse due to its capacity to induce dopamine release (12, 28). An alternative treatment option is methylphenidate, which has a lower potential for abuse compared to amphetamine; however, it still poses a noteworthy risks (29, 30). Developing safer treatment options with fewer adverse effects is therefore important and might be achieved by enhancing the serotonergic activity of substances that may exert an inhibitory effect on mesolimbic dopamine neurotransmission (31, 32). Small structural modifications can dramatically alter drug pharmacology, and investigating structure-activity relationships (SARs) can provide valuable insights to assist in medicinal chemistry and drug discovery.

The present study provides a pharmacological characterization of prolintane and six newly synthesized ring-substituted prolintane analogs using a variety of uptake inhibition and release assays in HEK293 cells expressing monoamine transporters and in rat brain synaptosomes. This study is aimed at adding to the current understanding of psychostimulant pharmacology.

## Results

### Methyl-substituted prolintane analogs show increased potencies at SERT relative to potencies at DAT and NET

**Figure 2:**
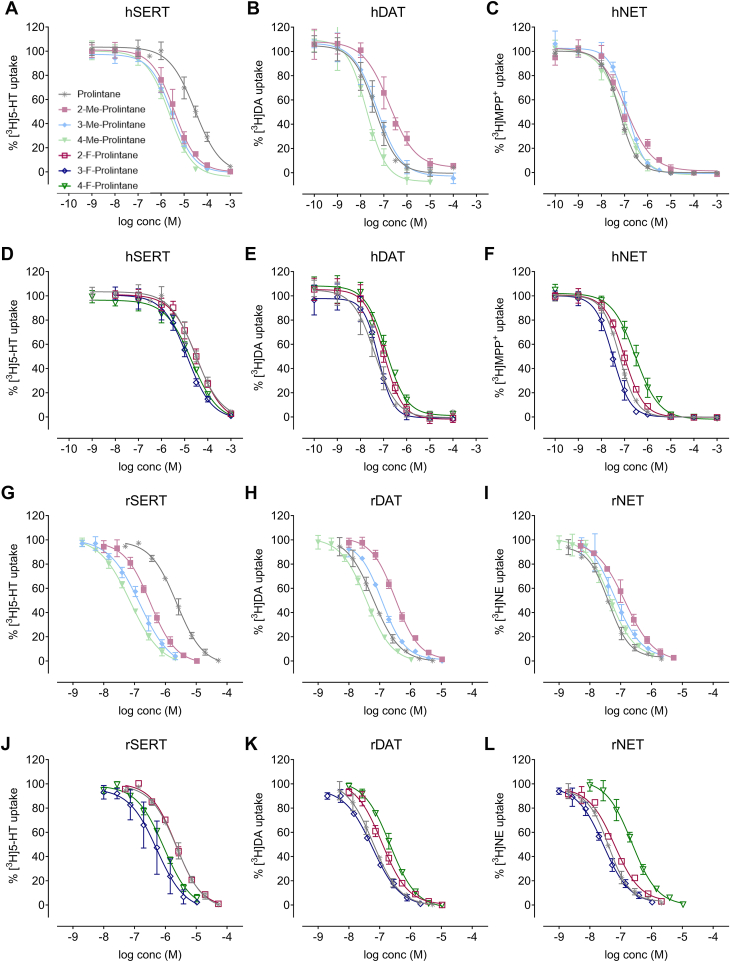
Uptake inhibition curves fitted with a sigmoidal dose–response of prolintane, 2-methylprolintane, 3-methylprolintane, 4-methylprolintane, 2-fluoroprolintane, 3-fluoroprolintane and 4-fluoroprolintane at (*A* and *D*) human serotonin transporter (hSERT), (*B* and *E*) human dopamine transporter (hDAT), (*C* and *F*) human norepinephrine transporter (hNET) transfected in HEK293 cells and (*G* and *J*) rat SERT (rSERT), (*H* and *K*) rat DAT (rDAT), (*I* and *L*) rat NET (rNET) in rat synaptosomes. Me, Methyl. F, Fluoro. Data is represented as mean ± standard deviation (SD) from three independent experiments performed in triplicate. IC_50_ values are represented in Table 1. Statistical analysis of IC_50_ values is included in the Supporting Material (Tables S3–S14).

**Figure 3:**
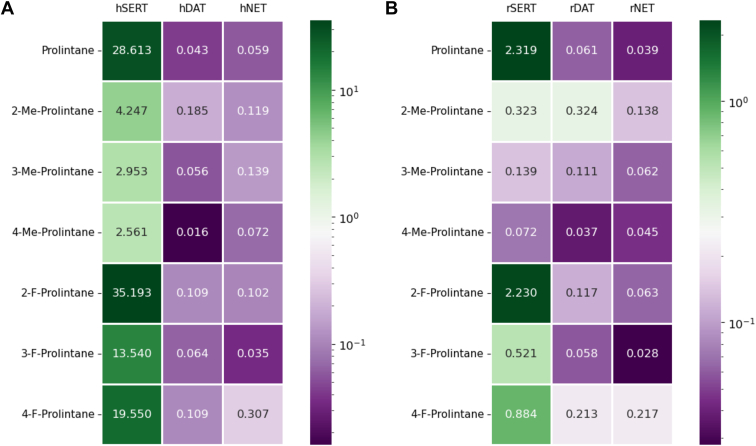
Heatmap showing IC_50_ values (μM) of prolintane, 2-methylprolintane, 3-methylprolintane, 4-methylprolintane, 2-fluoroprolintane, 3-fluoroprolintane and 4-fluoroprolintane at (*A*) human serotonin transporter (hSERT), human dopamine transporter (hDAT), human norepinephrine transporter (hNET) in transfected HEK293 cells and (*B*) rat serotonin transporter (rSERT), rat dopamine transporter (rDAT), rat norepinephrine transporter (rNET) in rat synaptosomes. Higher values (lower potency) are represented as *dark green*, lower values (higher potency) are represented as *dark purple* and signify a higher potency (see legend bar). Me, Methyl. F, Fluoro.

Prolintane is reported to interact with monoamine transporters of the SLC6 family, thus elucidating that the effects of prolintane analogs at these transporters is important for understanding the SAR for these compounds. Uptake inhibition assays can be used to determine whether substances interact with monoamine transporters. Concentration-response curves showing the potencies of prolintane and its analogs to inhibit uptake at hSERT, hDAT, and hNET are depicted in Fig. 2*A*–*F*, with corresponding IC_50_ values and calculated DAT/SERT ratios (${DAT/SERT}_{ratio}=\frac{1/{DAT}_{IC50}}{1/{SERT}_{IC50}}$) in Table 1. For better visualization of relative potencies across all drugs, IC_50_ values at human transporters are also displayed as a heatmap in Fig. 3*A*.

**Table 1:** IC_50_ values (μM) calculated from uptake inhibition assays for prolintane, 2-methylprolintane, 3-methylprolintane, 4-methylprolintane, 2-fluoroprolintane, 3-fluoroprolintane and 4-fluoroprolintane obtained from uptake inhibition assays represented in mean ± SD

Substance	Isoform	[^3^H]5-HT uptake SERT	[^3^H]DA uptake DAT	[^3^H]MPP^+^/[^3^H]NE uptake NET	DAT/SERT_ratio_
Prolintane	h	28.613 ± 4.012	0.043 ± 0.024	0.059 ± 0.007	670
	r	2.319 ± 0.169	0.061 ± 0.006	0.039 ± 0.004	38
2-Methylprolintane	h	4.247 ± 1.779	0.185 ± 0.065	0.119 ± 0.049	23
	r	0.323 ± 0.28	0.324 ± 0.025	0.138 ± 0.013	1
3-Methylprolintane	h	2.953 ± 0.541	0.056 ± 0.011	0.139 ± 0.033	53
	r	0.139 ± 0.014	0.111 ± 0.008	0.062 ± 0.009	1.25
4-Methylprolintane	h	2.561 ± 0.515	0.016 ± 0.002	0.072 ± 0.014	159
	r	0.072 ± 0.005	0.037 ± 0.003	0.045 ± 0.005	1.9
2-Fluoroprolintane	h	35.193 ± 9.249	0.109 ± 0.019	0.102 ± 0.022	323
	r	2.230 ± 0.152	0.117 ± 0.010	0.063 ± 0.006	19
3-Fluoroprolintane	h	13.54 ± 1.842	0.064 ± 0.02	0.035 ± 0.011	210
	r	0.521 ± 0.116	0.058 ± 0.004	0.028 ± 0.003	9
4-Fluoroprolintane	h	19.55 ± 2.426	0.109 ± 0.041	0.307 ± 0.097	179
	r	0.884 ± 0.078	0.213 ± 0.013	0.217 ± 0.021	4.2

h, human; r, rat; SERT, serotonin transporter; DAT, dopamine transporter; NET, norepinephrine transporter; 5-HT, serotonin; DA, dopamine; NE, norepinephrine.

${DAT/SERT}_{ratio}=\frac{1/{DAT}_{IC50}}{1/{SERT}_{IC50}}$

In the cell-based assays, we found that prolintane potently inhibits hDAT and hNET, consistent with prior findings (13). IC_50_ values for prolintane at hDAT and hNET were 0.043 ± 0.024 μM and 0.059 ± 0.007 μM, whereas the IC_50_ value at hSERT was more than 500-fold weaker at 28.613 μM. Substituting a methyl group at the 2-, 3-, or 4*-*position on the phenyl ring markedly and uniformly increased potency at hSERT (Fig. 2*A*), whereas 2-methyl substitution decreased potency at hDAT (Fig. 2*B*). Methyl ring substitutions did not substantially change potencies at hNET (Fig. 2*C*). Fluorine substitution of the phenyl ring had little effect on potency at hSERT or hDAT (Fig. 2*D* and *E*), but 2- and 4-fluoro analogs exhibited somewhat weaker potency at hNET (Fig. 2*F*). Overall, methyl ring substitution induced large reductions in hDAT/hSERT ratios, whereas fluorine substitution of the phenyl ring had weaker effects in this regard (see Table 1).

As a means to evaluate transporter activity of prolintane analogs in native tissue preparation, we carried out uptake inhibition experiments in rat brain synaptosomes. Concentration-response curves showing the potencies of prolintane and its analogs to inhibit uptake at rSERT, rDAT, and rNET are depicted in Figure 2*G*–*L*, with corresponding IC_50_ values and calculated rDAT/rSERT ratios in Table 1. IC_50_ values at rat transporters are also displayed as a heatmap in Figure 3*B*. In rat brain synaptosome assays, we found that prolintane potently inhibited rDAT and rNET, consistent with our findings in HEK293 cells and the work of others (13). IC_50_ values for prolintane at rDAT and rNET were 0.061 ± 0.006 μM and 0.039 ± 0.004 μM, whereas the IC_50_ value at rSERT was about 50-fold weaker at 2.319 ± 0.169 μM. Importantly, we found that potencies for prolintane and its analogs at rDAT and rNET were in the same range as those observed for hDAT and hNET (see Table 1). By contrast, drug potencies at rSERT were typically about 10 to 20-fold greater than those observed for hSERT, and this relationship was conserved across the various analogs. Substituting a methyl group at the 2-, 3-, or 4-position markedly increased potency at rSERT (Fig. 2*G*), leading to non-selective transporter blockers. Substituting a 2-methyl reduced potency at rDAT and rNET (Fig. 2*H* and *I*). Adding fluorine to the 3- or 4-position on the phenyl ring enhanced potency at rSERT (Fig. 2*J*), while 2- and 4-fluoro analogs exhibited weaker potencies at rDAT and rNET (Fig. 2*K* and *L*). Similar to the results with human transporters, methyl ring substitutions induced large decreases in rDAT/rSERT ratios, whereas fluoro ring substitutions had weaker effects in this regard (see Table 1).

### Prolintane and its substituted analogs show no release at hDAT and hNET but act as substrates and releasers at hSERT

The present uptake inhibition findings are consistent with published reports that show prolintane is a transporter inhibitor (13); however, the potential for prolintane or its analogs to induce transporter-mediated release has not been investigated. Uptake inhibition assays do not allow for differentiation between substances that are solely inhibitors vs. those that may also function as substrate-type releasers (9, 10, 11, 12). Thus, we examined transporter-mediated release in HEK cells transfected with either hDAT, hNET, or hSERT using batch release assays, collecting 2 min fractions after a pre-incubation with the radiolabeled substrate (33). The addition of a drug of interest at the IC_50_ concentration derived from uptake inhibition assays was used to assess efflux. Specificity of release was further evaluated by the addition of monensin, an ionophore that dissipates the sodium gradient across cell membranes (34). Monensin can be used to distinguish substrates from non-transported inhibitors, as it selectively enhances the efflux caused by transporter substrates (35, 36, 37).

**Figure 4:**
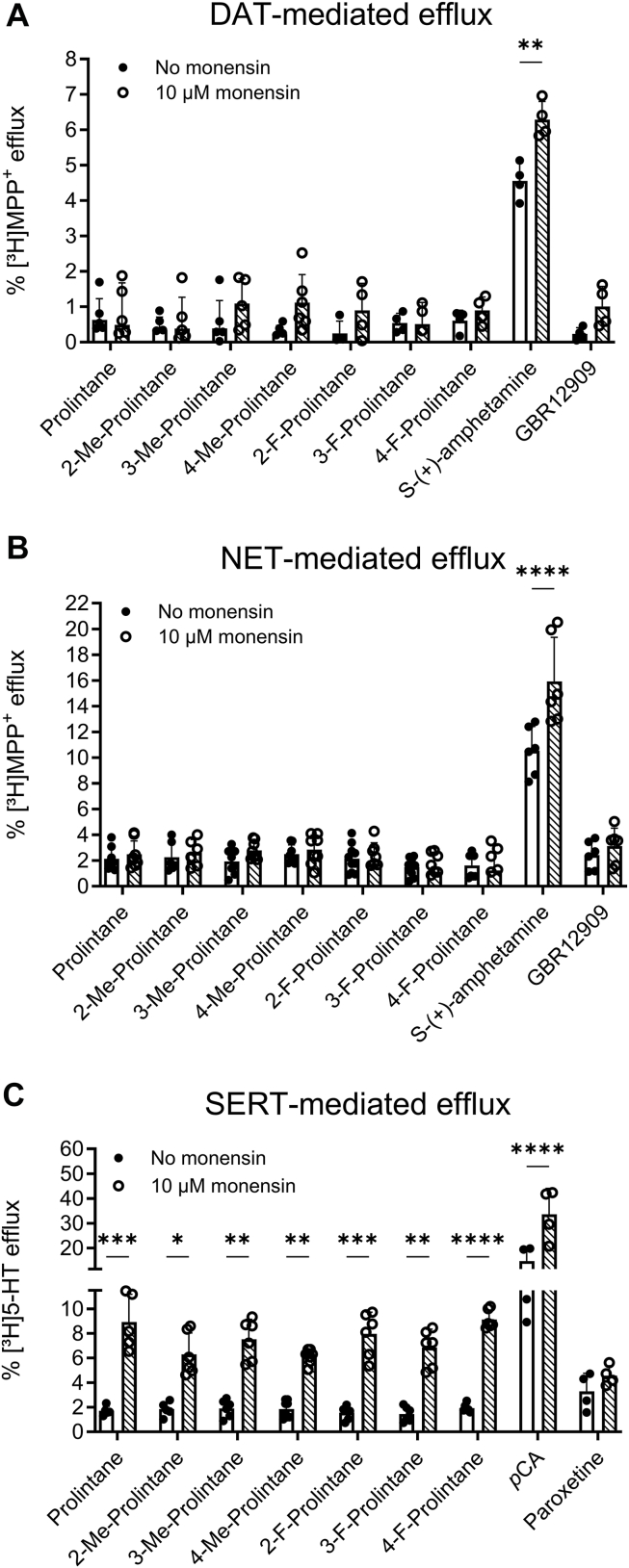
Transporter-mediated release (batch release assay) in transfected Hek293 cells of [^3^H]5-HT (human serotonin transporter; hSERT) or [^3^H]MPP^+^ (human dopamine/norepinephrine transporter; hDAT, hNET) expressed in % efflux of total uptake for prolintane, 2-methylprolintane, 3-methylprolintane, 4-methylprolintane, 2-fluoroprolintane, 3-fluoroprolintane and 4-fluoroprolintane at (*A*) hDAT, (*B*) hNET, and (*C*) hSERT. Compounds approximately at IC_50_ concentration (see Table 1) were added after establishing a baseline with and without the addition of monensin (Mon). Me, Methyl. F, Fluoro. Data is represented as mean ± standard deviation (SD) from two or three independent experiments (N = 2–3) performed in duplicate. Data was analyzed using mixed-effects analysis with factors of drug and Mon treatment, followed by Sidak's multiple comparisons test comparing each drug with and without Mon.∗p < 0.05; ∗∗p < 0.01, ∗∗∗p < 0.001, ∗∗∗∗p < 0.0001. Statistical tables are embedded in the Supporting Information (Tables S15–S20).

Figure 4*A* and *B* show no significant effect of prolintane or its analogs on efflux at hDAT and hNET, in the absence or presence of monensin. These findings support the notion that prolintanes act solely as inhibitors at hDAT and hNET. A positive control releaser, *S*-(+)-amphetamine, and a negative control inhibitor, GBR12909, were also tested to ensure the integrity of the assay. In contrast to the findings with hDAT and hNET, Figure 4*C* demonstrates that prolintane and its analogs produced small increases in SERT-mediated efflux that were markedly enhanced in the presence of monensin. The findings with hSERT suggested that prolintanes are capable of inducing transporter-mediated release of 5-HT.

**Figure 5:**
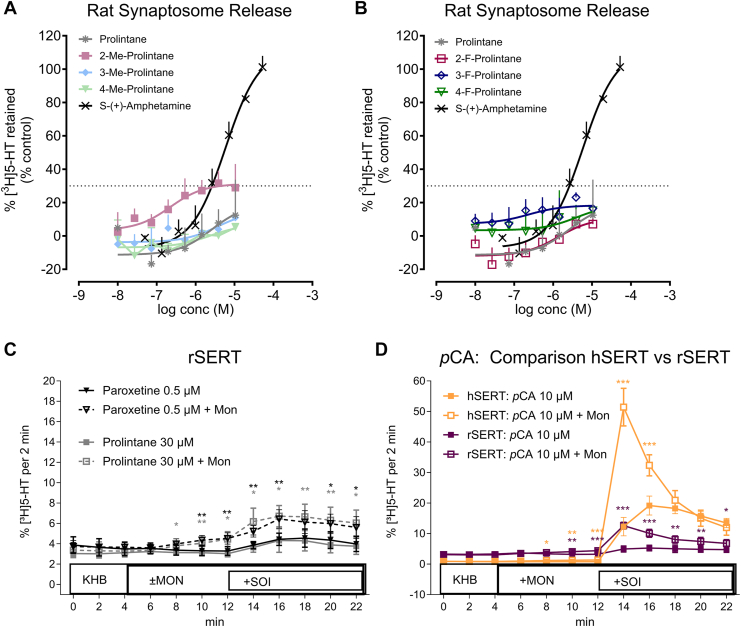
Concentration-dependent serotonin transporter (SERT) mediated efflux of prolintane, S-(+)-amphetamine, 2-methylprolintane, 3-methylprolintane, 4-methylprolintane (*A*), 2-fluoroprolintane, 3-fluoroprolintane and 4-fluoroprolintane (*B*) in rat synaptosomes. Transporter-mediated release of (*C*) prolintane, paroxetine at rSERT and (*D*) para-chloroamphetamine (pCA) at rSERT *versus* hSERT using superfusion assays in transfected HEK293 cells. Time-dependent [^3^H]5-HT efflux elicited by addition of the substance of interest (SOI). Compounds were added after establishing a baseline by superfusing with Krebs-HEPES buffer (KHB) with and without the addition of monensin (Mon). Data is represented as mean ± standard deviation (SD) from three independent experiments (N = 3) performed in triplicate. Time-course data was analyzed using two-way repeated measures ANOVA with factors of time and Mon treatment, followed by Sidak's multiple comparisons test to assess differences at individual time points. ∗p < 0.05; ∗∗p < 0.01; ∗∗∗p < 0.001. Statistical tables are embedded in the Supporting Information (Tables S37–S44). Me, Methyl. F, Fluoro. 5-HT, serotonin.

As a means to verify the SERT-mediated efflux of 5-HT in a native tissue preparation, we tested the effects of prolintane and its analogs using transporter release assays in rat brain synaptosomes. Full concentration-response curves from 1 nM to 10 μM for prolintane and each of its analogs revealed no evidence for robust transporter-mediated efflux at rSERT (Fig. 5*A* and *B*). In the rat synaptosome assays, compounds with releasing efficacy <30% of maximal are considered inactive, since non-transported inhibitors like paroxetine induce low efficacy “pseudo-efflux” to about this level. Collectively, the findings suggest that prolintanes are pure uptake inhibitors at rat transporters, but they act as hybrid compounds at human transporters, whereby they act as inhibitors at hDAT and hNET but substrates at hSERT. To better understand the different releasing effects of prolintanes at hSERT *versus* rSERT, we performed additional release assays in HEK293 cells transfected with rSERT. We found that prolintane and the SERT inhibitor paroxetine share an almost identical pseudo-efflux releasing profile in cells expressing rSERT (Fig. 5*C*), and this efflux response was not enhanced by monensin. Additionally, the known monoamine releaser, para-chloroamphetamine (*p*CA), induced a significantly lower magnitude of [^3^H]5-HT release in cells expressing rSERT when compared to its effects in cells expressing hSERT (Fig. 5*D*), consistent with the literature (38).

**Figure 6:**
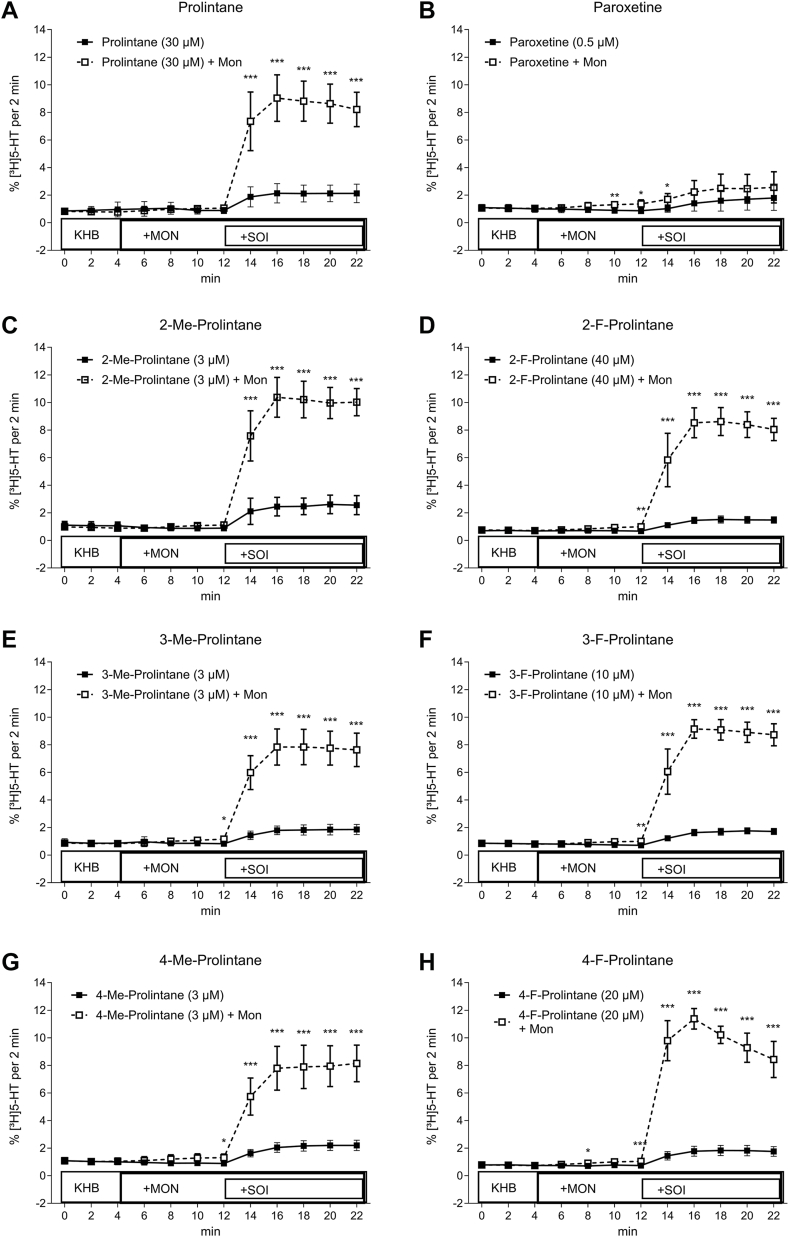
**Transporter-mediated release of prolintane using superfusion assays in transfected HEK293 cells.** Time-dependent [^3^H]5-HT efflux elicited by addition of the substance of interest (SOI) (*A*) prolintane, (*B*) paroxetine, (*C*) 2-methylprolintane, (*E*) 3-methylprolintane, (*G*) 4-methylprolintane, (*D*) 2-fluoroprolintane, (*F*) 3-fluoroprolintane, and (*H*) 4-fluoroprolintane at human serotonin transporter (hSERT). Compounds were added in IC_50_ concentrations derived from uptake inhibition assays (indicated in each panel) after establishing a baseline by superfusing with Krebs-HEPES buffer (KHB) with and without the addition of monensin (Mon). Me, Methyl. F, Fluoro. Data is represented as mean ± standard deviation (SD) from three independent experiments (N = 3) performed in triplicate. Time-course data were analyzed using two-way repeated measures ANOVA with factors of time and Mon treatment, followed by Sidak's multiple comparisons test to assess differences at individual time points. ∗p < 0.05; ∗∗p < 0.01; ∗∗∗p < 0.001. Statistical tables are embedded in the Supporting Information (Tables S21–S36).

Given the unique species differences in the activity of prolintanes at SERT, we carried out additional experiments in cells transfected with hSERT to further verify 5-HT-releasing actions. Figure 6 shows the effects of prolintane and its analogs on hSERT-mediated release under superfusion conditions, which affords increased sensitivity compared to the batch release assay. In the absence of monensin, prolintanes caused small increases in 5-HT release. However, in the presence of monensin, the weak releasing activity at hSERT was dramatically enhanced for all the prolintanes. All compounds were tested at their respective IC_50_ values derived from uptake inhibition experiments (see Fig. 2). Methyl-substituted analogs (Fig. 6, *C*, *E* and *G*) showed release at lower tested concentrations due to their higher potency in uptake inhibition assays, whereas fluorine-substituted analogs (Fig. 6, *D*, *F* and *H*) were tested at higher concentrations and showed effects similar to prolintane (Fig. 6*A*). Importantly, since each compound was only tested at a single concentration, no conclusions can be drawn regarding their maximal efficacy or release potency.

**Figure 7:**
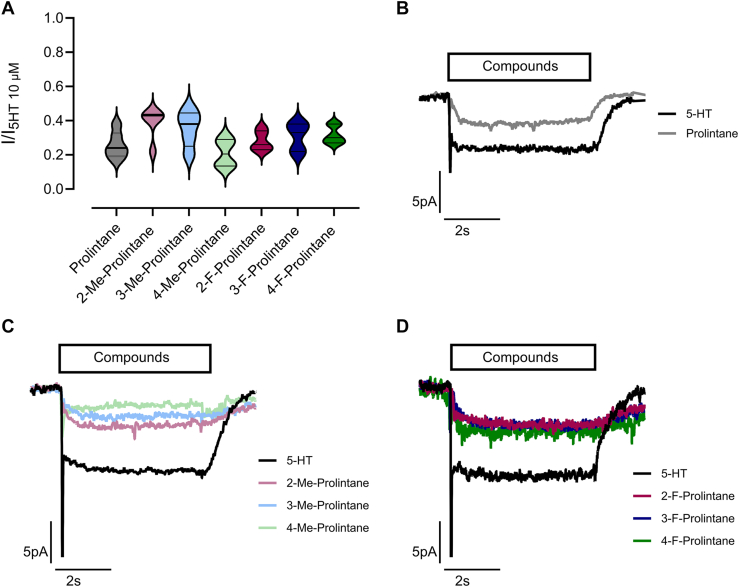
**Human serotonin transporter (hSERT) mediated inwardly directed currents in HEK293 cells detected by whole-cell patch clamp experiments (N = 5).***A*, steady state currents normalized to currents elicited by application of 10 μM serotonin (5-HT). *B–D*, representative single-cell traces showing currents elicited by uptake inhibition IC_50_ concentrations of prolintane and its analogs. Descriptive statistics are embedded in the Supporting Information (Table S45).

We next performed electrophysiological patch-clamp measurements using the whole-cell configuration in HEK293 cells overexpressing hSERT to provide additional insights into the substrate-type releasing properties of the compounds (39). Since hSERT utilizes the Na^+^ gradient to co-transport 5-HT and other substrates, the application of a substrate induces a sustained inward-directed current that remains throughout the exposure (40, 41). The current-inducing property of a given compound is commonly used to determine whether a test compound can function as a substrate (42, 43). Application of prolintane and its analogs at IC_50_ concentrations led to steady-state currents of 20 to 40% of those elicited by 10 μM 5-HT (Fig. 7*B*–*D*), indicating that the substances get translocated across the plasma membrane and therefore act as substrates at hSERT. However, it is important to note that the magnitude of inward current induced by prolintanes was less than that of 5-HT (Fig. 7*A*), suggesting prolintanes are partial substrates of hSERT.

We performed control experiments in HEK293 cells overexpressing hSERT by adding the selective serotonin inhibitor paroxetine, which completely abolished prolintane-induced current (Fig. S2). Furthermore, in native HEK293 cells without transfection, no current could be recorded for 5-HT or prolintane (Fig. S3). These findings verify that the recorded currents were indeed mediated through hSERT.

**Figure 8:**
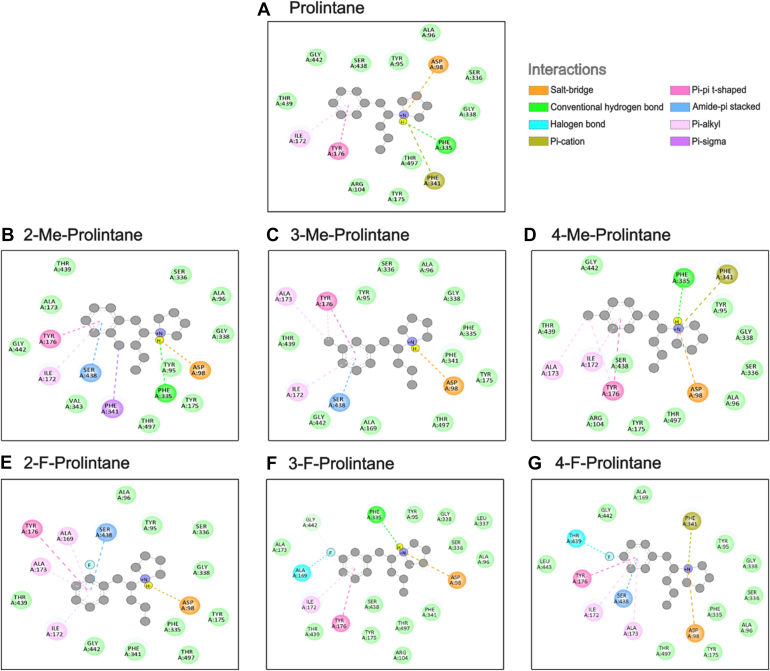
**2D interaction diagrams of hSERT–prolintanes complexes.** The ligand is depicted as a disc model in *grey*, with surrounding amino acid residues labelled with residue name, chain ID, and residue number. Interaction types are indicated by *dashed lines* and color-coded according to the legend at the *top right*. Each diagram represents the selected binding pose of (*A*) prolintane, (*B*) 2-methylprolintane, (*C*) 3-methylprolintane, (*D*) 4-methylprolintane, (*E*) 2-fluoroprolintane, (*F*) 3-fluoroprolintane, and (*G*) 4-fluoroprolintane.

**Figure 9:**
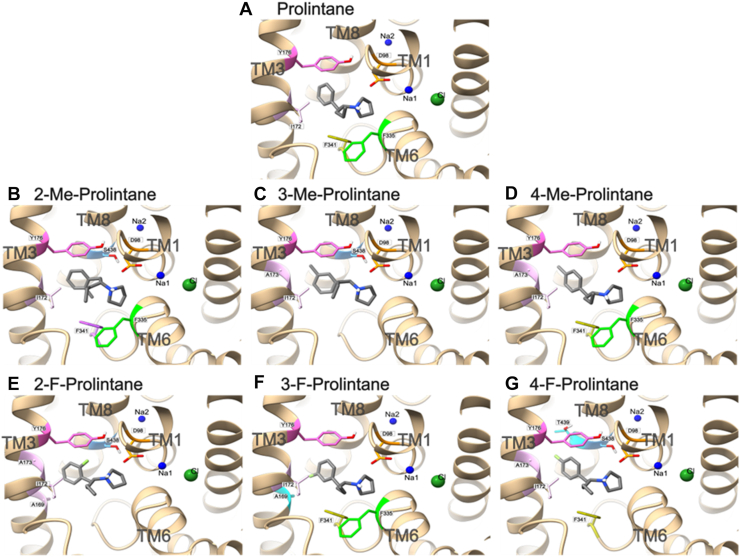
**3D binding site interactions of hSERT-prolintanes complexes.** Each panel (*A–G*) shows the selected binding pose of docked ligand (*grey sticks*) within the hSERT (*tan cartoon*) binding pocket. Sodium (Na1, Na2) and chloride (Cl^−^) ions are shown as *blue* and *green spheres*, respectively. Binding site residues are labelled and shown in stick representation with their side chains highlighted in the same colors used in the 2D interaction diagrams.

To understand the substrate profile of prolintanes, we performed molecular docking of drug binding poses and their interactions within the S1 binding site of hSERT. We aimed to identify poses that have spatial similarity with 5-HT in the cryo-EM structure (PDB ID: 7LIA), specifically the placement of the nitrogen atom and phenyl ring. The selected binding poses of prolintanes exhibited interaction profiles similar to the exogenous substrate 5-HT (Figs. 8 and 9). The binding poses obtained from molecular docking revealed that the positively charged nitrogen atom of prolintanes forms a salt bridge with the carboxylate of the conserved D98. Moreover, hSERT-prolintane interactions include the phenyl ring of prolintanes forming a hydrophobic interaction and π–π stacking in the hydrophobic sub-pocket of the S1 with I172 and Y176, respectively.

## Discussion

The primary aim of this study was to evaluate the interactions of prolintane and six of its analogs with the SLC6 monoamine transporters, SERT, DAT, and NET. The investigation of prolintanes is important since these compounds share common structural features with pyrrolidine-containing cathinones (*e.g.*, α-PVP) that are problematic drugs of abuse. Prior studies demonstrated that prolintane acts as a potent inhibitor at hDAT and hNET, with approximately 10-fold weaker effects at hSERT (13). The present findings with human transporters generally agree with the work of Eshelman *et al.* (2019), but the hSERT potencies that we observed here are an order of magnitude lower than those reported previously. The present findings with rat transporters are more consistent with Eshleman *et al.* (2019) demonstrating potent inhibition at rDAT and rNET, with 10-fold lower potency at rSERT. Our data are the first to show that prolintane phenyl ring substitution at the 2-, 3- or 4-position increases potency at SERT relative to DAT, and this is especially true for methyl ring substitutions. Finally, the present data reveal that prolintane and its analogs can function as substrates at hSERT and can induce 5-HT efflux. Thus, prolintanes can be categorized as “hybrid” transporter compounds at human transporters, whereby the compounds act as pure inhibitors at hDAT and hNET but substrates at hSERT (41).

It is important to reiterate that prolintane and its analogs act as weak partial releasers at hSERT (44, 45), since the magnitude of efflux is much less than full efficacy releasers such as *p*CA (44, 46). Different theories have been proposed to explain partial releasing efficacy, including slow binding kinetics at the transporter, reduced rate of hSERT-mediated efflux, or binding to allosteric sites on the transporter (21, 42, 43, 47). We performed molecular docking to better understand how prolintane and its analogs can act as substrates at hSERT, since it was an unexpected finding based on previous publications (41). The binding poses obtained from molecular docking showed a salt bridge formation between the carboxylate of the conserved D98, which is an important residue for substrate recognition and translocation (48, 49), and the positively charged nitrogen atom of prolintane and its analogs. This salt bridge formation mimics the interactions of hSERT with 5-HT, although there were subtle differences in interactions among poses for the prolintanes, such as hydrogen bonding with F335 or contacts with F341, S438, A169, and A173. Nevertheless, these slight variations in docking poses for prolintanes do not appear to disrupt the conserved binding pose, and could reflect limitations in docking precision, the cutoff used in the analysis, or flexibility in how the ligands are accommodated within the static model of the binding site. The similar binding poses for 5-HT and the prolintanes imply that the compounds can interact with the necessary residues for translocation, supporting their substrate-like behavior in functional assays.

It is noteworthy that prolintanes induced 5-HT efflux through hSERT but not rSERT. At present, we have no explanation for the species difference in 5-HT releasing activity for the compounds. One possibility is that prolintanes interact somewhat differently at human vs. rat isoforms of SERT, whereas other explanations include differences in the methods used to measure SERT-mediated release in transfected cells vs. brain synaptosomes. Generally, transporter ligands displaying <30% of maximal release are considered inactive in synaptosome release assays, because uptake inhibitors can evoke this degree of partial efflux. Our present findings suggest that rat synaptosome assays are less sensitive than cell-based assays for evaluating transporter-mediated efflux, since the potential releasing properties of prolintanes could not be easily discerned in synaptosomes. By contrast, the experiments in HEK293 cells transfected with rSERT revealed that paroxetine and prolintane evoke a similar degree of pseudo-efflux that is not altered by monensin, showing that prolintanes likely act as pure inhibitors at rSERT. Notably, the maximal efflux of [^3^H]5-HT induced by *p*CA was significantly lower in rSERT-expressing cells than in hSERT-expressing cells, leading to a small effect size window for rSERT. This reduced efflux of [^3^H]5-HT at rSERT was shown previously (38), and highlights a key species difference observed for the effects of transporter ligands. In any case, more research is needed to clarify the nature of species differences in the activity of prolintanes and other substances at SERT.

When compared to its structural analog α-PVP, prolintane shows lower potency at DAT and NET for both human and rat transporters (13, 17, 18, 19, 44). Moreover, prolintanes have increased potency at SERT when compared to α-PVP, engendering lower DAT/SERT ratios. High DAT/SERT ratios are presumed to indicate greater abuse liability for transporter ligands (22, 50, 51, 52). 4-Methylprolintane exhibited four-fold greater inhibitory potency at hDAT but a 10-fold greater inhibitory potency at hSERT compared to its β-keto analogue pyrovalerone (53), leading to a decreased hDAT/hSERT ratio. When comparing α-PVP and pyrovalerone, the methyl substitution similarly increases potency at SERT, as it does for prolintane (20). 2-, 3- and 4-Methylprolintanes acted as non-selective uptake inhibitors at rat transporters, with rDAT/rSERT ratios close to unity. With α-PVP, 3- and 4-position ring substitution with fluorine did not result in significant changes in potency at hDAT (23). In general, ring substitutions for amphetamines, cathinones, ephedrine or α-PVP lead to increases in serotonergic activity (44, 53, 54, 55), similar to the ring-substituted prolintane analogs shown in this study. For α-pyrrolidinopropiophenone (α-PPP), substitution with a halogen led to increasing interaction with hSERT (44) compared to methyl substitution, while other authors described similar effects for methyl or halogen substituted cathinones (53, 56). We found that methylprolintanes showed greater inhibition potency at hSERT when compared to fluoroprolintanes.

With regard to the limited clinical data with prolintane, there appear to be dose-dependent effects that range from stimulation and euphoria, sympathomimetic effects, reductions in total REM sleep, insomnia, and even hallucinations (1, 14, 15, 16). Lee *et al.* recently described rewarding and reinforcing properties for moderate to high doses of prolintane in mice. However, when compared to cocaine and methamphetamine, larger prolintane doses were needed to induce these effects (2).

Prolintane was regularly used as a stimulant for a number of therapeutic indications, including ADHD-like symptoms and narcolepsy, from the 1950s to 2000s (1, 2). Current ADHD treatment strategies primarily involve amphetamine or methylphenidate, both of which present an inherent risk for potential misuse. (12, 28, 29, 30). The novel prolintane analogs described here may serve as a valuable starting point for the development of new candidates for ADHD treatment, as they were inactive as releasers at DAT and NET, suggesting a low potential for abuse and cardiovascular stimulation, and they showed lower DAT/SERT ratios. Further investigations should include *in vivo* experiments to test the rewarding and reinforcing effects of prolintanes, including conditional place preference paradigms and self-administration studies. At the same time, experiences gained from the NPS phenomenon (24) demonstrate how information regarding potential drug candidates can enter the recreational market. Our findings underscore the importance of conducting pharmacological investigations with novel stimulants, as the resulting data can be valuable to professionals working in the fields related to law enforcement, healthcare and policymaking.

In conclusion, we found that: i) prolintane and the six phenyl-substituted analogs are potent inhibitors of DAT and NET, with weaker effects at SERT, ii) ring-substituted prolintane analogs show reduced DAT/SERT ratios compared to prolintane, with more robust effects for methyl ring substitutions, and iii) prolintane analogs act as weak partial substrates and releasers at hSERT but not rSERT. Future studies should explore the *in vivo* pharmacological effects of prolintane analogs to determine their potential adverse and therapeutic attributes.

## Experimental procedures

### Drugs and reagents

Starting materials, reagents and solvents used for synthesis were obtained from Sigma Aldrich, Alfa Aesar or Oakwood Chemical or 1PlusChem or VWR. Column chromatography was conducted using Merck silica gel (Sigma Aldrich, St Louis, MO, USA), grade 9385 (230–400 mesh). Drugs used as specific blockers or releasers were bought from different companies: vanoxerine (GBR12909), dextroamphetamine hemi sulfate salt (*S*-(+)-amphetamine), monensin were purchased from Sigma-Aldrich, while paroxetine HCl was purchased from abcr GmbH. Cell culture supplies were obtained from Capricorn Scientific GmbH, including Dulbecco's Modified Eagle Medium (DMEM) high glucose (4.5 g/L) with L-glutamine, fetal bovine serum, geneticin (G418; 50 mg/ml; #G418-B). Radiotracer compounds were ordered from Revvity (Waltham, MA, USA), including [^3^H]5-HT (1 mCi), [^3^H]DA (1 mCi), [^3^H]NE (1 mCi), and [^3^H]1-methyl-4-phenylpyridinium ([^3^H]MPP^+^; 250 μCi).

### Instrumentation

#### Melting point determination

Melting points were determined using a MPA100 Melting Point Apparatus (Stanford Research Systems) at a ramp rate of 2 °C/min.

#### Nuclear Magnetic Resonance spectroscopy

^1^H NMR (400 MHz) and ^13^C (101 MHz) spectra were obtained on 20 mg/ml solutions of thehydrochloride salts in anhydrous d_6_-DMSO (>99.9% D, Sigma Aldrich) on a Bruker Ultrashield 400 plus spectrometer with a 5 mm BSO S1 (Z gradient plus) probe at ambient temperature. Internal chemical shift references were solvent (δ = 2.5 and 39.52 ppm for ^1^H and ^13^C spectra, respectively). ^1^H and ^13^C NMR assignments were made using ^1^H and ^13^C (PENDANT) chemical shift position, splitting pattern (^1^H), and hetero- and homo- 2-D experiments including homonuclear correlation spectroscopy (COSY), heteronuclear multiple quantum coherence (HMQC), and heteronuclear multiple bond coherence (HMBC).

#### High-performance liquid chromatography (HPLC)

An Agilent 1260 Infinity system was used that is comprised of a 1260 quaternary pump VL, a 1260 ALS autosampler, a 1260 Thermostatted Column Compartment, and a 1200 DAD Multiple Wavelength Detector (Agilent Technologies, CA, USA). A detection wavelength of 220 nm was used to estimate purity. A Zorbax Eclipse Plus-C18 analytical column (5 μm, 4.6 × 150 mm) from Agilent (Agilent Technologies) was used for chromatographic separation of the compounds. Mobile phase A consisted of a 10 mM aqueous ammonium formate buffer prepared in HPLC-grade water, titrated to pH 4.5 using a 10 mM formic acid solution. The buffer was prepared the day of the experiments. Mobile phase B consisted of HPLC-grade acetonitrile (ACN). Compound samples were prepared at a concentration of 2.0 mg/ml. An injection volume of 40 μl was used, the flow rate was 1.0 ml/min, and the column temperature was set to 25 °C (ambient temperature). Runs were isocratic with a solvent ratio of 7:3 A:B. and run for 10 min, collected at 220 nm. Chromatograms (retention times and peak areas) were analyzed using Agilent ChemStation Software (Agilent Technologies).

#### High Resolution mass Spectrometry (HRMS)

HRMS experiments were performed using a Thermo Orbitrap Exactive Mass Spectrometer with an Orbitrap mass analyzer in positive mode. The instrument was calibrated using electrospray ionization with Pierce LTQ ESI Positive Ion Calibration Solution (ThermoFisher Scientific). Samples (HCl salt) were analyzed using an Atmospheric Solids Analysis Probe (ASAP) source. Data analysis was performed on Thermo Xcalibur Qual Browser software, and compound formulas were confirmed if the observed m/z was <5.0 ppm error from the theoretical. Measurement parameters: Aux gas flow rate-8, Spray Voltage-3.50 kV, Capillary temperature 275 °C, Capillary Voltage 25.00 V, Tube Lens Voltage 65.00 V, Skimmer Voltage 14.00 V, Heater Temperature 100 °C.

### Chemical syntheses

**Figure 10:**
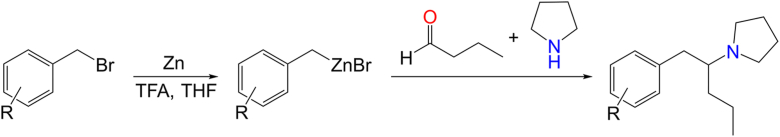
Synthetic route utilizing a modified One-Pot Mannich Barbier reaction to produce prolintane and analogs.

Target compounds were synthesized *via* a novel route consisting of a modified one-pot Mannich Barbier reaction (57) (Fig. 10). The deviation from the prior published method involves the preformation of the benzyl zinc reagent (non-Barbier conditions) and modification of the work up conditions. These changes were observed to consistently improve yields, especially when using an aliphatic aldehyde, as in this case. Detailed syntheses are provided in the supporting information. The structure of synthesized compounds and related compounds is depicted in Figure 1.

### Cell culture

HEK293 cells were stably transfected with the human isoform (h) of the transporter of interest and kept at 37 °C in a humidified atmosphere, as described in detail previously (58). HEK293 cells were last authenticated in April 2024 by CLS Cell Lines Service GmbH, Eppenheim, Germany, using short tandem repeat profiling. Cells were cultured in Dulbecco's modified Eagle's medium (DMEM) supplemented with 10% fetal bovine serum and 250 μg/ml geneticin, 6 μg/ml blasticidin and/or 120 μG/ml zeocin for selection maintenance.

### Uptake inhibition assays in transfected cells

HEK293 cells stably expressing human monoamine transporters hSERT, hDAT or hNET were seeded into poly-D-lysine (PDL) coated 96-well plates with a density of approximately 36,000 cells per well 1 day prior to experiments. A day after seeding, the cell medium was replaced with 200 μl Krebs-HEPES-buffer (KHB; 120 mM NaCl, 3 mM KCl, 2 mM CaCl_2_·2H_2_O, 2 mM MgCl_2_·6H_2_O, 20 mM D-glucose, pH = 7.3). The test drugs were initially dissolved in Milli-Q H_2_O or dimethyl sulfoxide (DMSO) at a concentration of 10 to 100 mM. Then, cells were treated with the respective concentration of the test drug diluted in KHB for 5 min as a pre-incubation step. Subsequently, in the uptake step, the solution was replaced with 50 μl/well diluted drug solution and tritiated substrate (hDAT: 0.1 μM [^3^H]DA; hNET: 0.02 μM [^3^H]MPP^+^; hSERT: 0.1 μM [^3^H]5-HT) for 1 min (hDAT, hSERT) or 3 min (hNET). After incubation, cells were immediately washed with 200 μl KHB to stop uptake. Uptake was indirectly measured by quantifying beta emission with liquid scintillation counting (Wallac 1450 MicroBeta TriLux Liquid Scintillation Counter & Lumi; GMI). Therefore, 200 μl Ultima Gold XR scintillation cocktail (PerkinElmer, MA, USA) was added to each well. The uptake in the absence of compound was defined as 100% and uptake in the presence of a known inhibitor (hDAT, hNET: 50 μM GBR12909; hSERT: 3 μM paroxetine) was defined as unspecific uptake (0%) and subtracted from each value.

### Batch release assay in transfected cells

Batch release assays were performed as described previously (44). In brief, HEK293 cells stably expressing monoamine transporters were seeded similar to the procedure used for uptake inhibition assays. A day after seeding cells were pre-loaded with 50 μl/well 0.015 μM [^3^H]MPP^+^ (hDAT, hNET), or 0.08 μM [^3^H]5-HT (hSERT) for 20 min at 37 °C. Then cells were washed four times for 3 to 4 min each and further incubated with KHB or KHB + monensin. After 10 min of incubation, two (screening) to four (full traces; hSERT) 2 min incubations were performed to establish a baseline release without substance. Subsequently, the test drugs were added at IC_50_ concentrations (determined from uptake experiments) with or without monensin for three (screening) to five (full traces; hSERT) consecutive 2 min incubation steps. Each fraction was transferred to an empty well in the 96-well plate. For screening, a known inhibitor (hDAT, hNET: 50 μM GBR12909) and releaser (hDAT, hNET: 10 μM *S*-(+)-amphetamine) were used as controls. Ultima Gold XR scintillation cocktail was added to the remaining cells, and each well contained a release fraction. The counted radioactivity in the respective fraction was expressed as a percentage of total radioactivity at the start of the fraction.

### Superfusion assay in transfected cells

A day prior to the experiment, HEK293 cells stably expressing hSERT or rat SERT (rSERT) were seeded at a density of 100,000 cells per channel into 6 channel flow ibidi slides (Gräfelfing, Germany). On the day of the experiments, cells were preloaded with 0.1 μM [^3^H]5-HT at 37 °C, 5% CO_2_ for 20 min. Subsequently, the slides were transferred to the superfusion apparatus (59). Cells were then washed with KHB for 15 min under a constant flow of 0.5 ml/min. Fractions were collected every 2 min in 8 ml vials containing 2 ml scintillation cocktail. Collection began with three baseline fractions using KHB, followed by four additional basal release fractions with KHB, either with or without 10 μM monensin. Subsequently, the test compound was perfused at its previously determined IC_50_ concentration, and five fractions were collected. To assess residual radioactivity within the cells, they were lysed with 1% sodium dodecyl sulfate (SDS), and three final fractions were obtained. The measured tritiated substrate in each fraction was expressed as a percentage of the initial tritiated substrate present at the start of that fraction.

### Patch-clamp electrophysiology in transfected cells

One day prior to recording, either native HEK293 cells or HEK293 cells stably expressing monoamine transporters were seeded at low density onto PDL-coated 35 mm dishes. Patch-clamp experiments were conducted at a holding potential of −60 mV, with substrate-induced currents recorded at room temperature (20–24 °C) using an Axopatch 700B amplifier and pClamp 11.2 software (MDS Analytical Technologies). During recordings, cells were continuously superfused *via* a DAD-12 perfusion system and an 8-tube manifold (ALA Scientific Instruments), delivering an external solution composed of 140 mM NaCl, 2.5 mM CaCl_2_, 3 mM KCl, 2 mM MgCl_2_, 20 mM glucose, and 10 mM HEPES, with pH adjusted to 7.3 using NaOH. The intracellular pipette solution, designed to mimic physiological ionic conditions, contained 133 mM potassium gluconate, 5.9 mM NaCl, 1 mM CaCl_2_, 0.7 mM MgCl_2_, 10 mM HEPES, and 10 mM EGTA, with pH set to 7.2 using KOH. Currents were filtered at 1 kHz and digitized at 10 kHz *via* a Digidata 1550 digitizer (MDS Analytical Technologies), followed by analysis in Clampfit 10.2 (Molecular Devices). Substance-induced currents were normalized to the current elicited by a saturating concentration of 5-HT (10 μM) of the same cell. Holding currents were subtracted, and a 50 Hz digital Gaussian low-pass filter was applied to the traces for further analysis.

### Statistical analysis for cell-based assays

Half-maximal inhibitory concentrations (IC_50_) were obtained from fitting a sigmoidal dose–response ($Y=\text{Bottom}+\frac{\text{Top}-\text{Bottom}}{1+10^{\left(\left(\text{LogIC}50-X\right)\cdot \text{HillSlope}\right)}}$) in GraphPad Prism 10.3.1 (GraphPad Software Inc.). Uptake inhibition data were evaluated by one-way ANOVA followed by Tukey's multiple comparisons test. Release data were analyzed using a two-way ANOVA (batch release) or a mixed-effects model with Geisser-Greenhouse correction (time-dependent traces) employing Šidák correction for comparing differences between compounds with or without the addition of monensin.

### Molecular docking

The outward-open hSERT structure was obtained from the Protein Data Bank (PDB ID: 7LIA (48)) and prepared by removing antibody fragments and co-resolved ligands except for the co-transported ions. The ligands, including prolintane and its analogs were built using Avogadro, and protonated at pH 7.3 by using Open Babel's functionality within Avogadro (60). The ligands were then energy minimized using the default parameters provided by Avogadro to obtain the optimized geometries prior docking. The docking grid box was defined to encompass the central binding site (S1) binding site of hSERT, ensuring that all relevant binding interactions could be evaluated, and docking calculations were performed using AutoDock Vina (61). BIOVIA Discovery Studio Visualizer was used to create 2D interaction diagrams of protein-ligand complexes (BIOVIA, Dassault Systèmes, v25.1.0.24284).

### Animals for the synaptosome experiments

For the transporter assays carried out in rat brain synaptosomes, 24 adult male Sprague-Dawley rats (Envigo, Frederick, MD, USA) were used. Rats weighing 250 to 300 g were group housed at the National Institute on Drug Abuse (NIDA) Intramural Research Program (IRP) animal facility in a temperature (22.2 ± 1.1 °C) and humidity (45 ± 10%) controlled room on a 12 h light-dark cycle (lights on at 0700) with free access to food and water. All animal procedures were reviewed and approved by the Institutional Animal Care and Use Committee of the NIDA IRP and were carried out in accordance with the Guide for the Care and Use of Laboratory Animals (National Research Council of the National Academies, 2011).

### Synaptosome preparation

Rats were killed by CO_2_ narcosis and decapitated, then synaptosomes were prepared from brains using standard procedures. In brief, synaptosomes were prepared from caudate tissue for rat DAT (rDAT) assays, or from whole brain minus caudate and cerebellum for rat NET (rNET) and rat SERT (rSERT) assays. Brain tissue was homogenized in ice-cold 10% sucrose followed by centrifugation at 1000*g* for 10 min. The resulting supernatant containing crude synaptosomes was kept on ice until use in uptake or release procedures. Uptake inhibition and release assays were carried out as previously described (62, 63).

### Uptake inhibition assays in synaptosomes

For uptake inhibition assays, 5 nM [^3^H]dopamine, 10 nM [^3^H]norepinephrine, or 5 nM [^3^H]5-HT was used as the radiolabeled transmitter for rDAT, rNET, or rSERT, respectively. To optimize uptake for a single transporter, unlabeled blockers were included that prevented uptake of [^3^H]transmitter by competing transporters: 50 nM GBR 12935 was added to NET and SERT assays to block DAT, 100 nM nomifensine was added to SERT assays to block NET. Uptake assays were initiated by adding 100 μl of tissue to 900 μl Krebs-phosphate buffer (126 mM NaCl, 2.4 mM KCl, 0.83 mM CaCl_2_, 0.8 mM MgCl_2_, 0.5 mM KH_2_PO_4_, 0.5 mM Na_2_SO_4_, 11.1 mM glucose, 0.05 mM pargyline, 1 mg/ml bovine serum albumin, and 1 mg/ml ascorbic acid, pH 7.4) containing test drug and [^3^H]transmitter. Uptake assays were terminated by rapid vacuum filtration, and retained radioactivity was quantified with liquid scintillation counting.

### Release assays in synaptosomes

For release assays, 9 nM [^3^H]MPP^+^ was used as the radiolabeled substrate for rDAT and rNET, whereas 5 nM [^3^H]serotonin ([^3^H]5-HT) was used for rSERT. All buffers used in the release assay contained 1 μM reserpine to block vesicular uptake of substrates. The selectivity of release assays was optimized for a single transporter by including unlabeled blockers to prevent the uptake of [^3^H]MPP^+^ or [^3^H]5-HT by competing transporters: 100 nM desipramine was added to DAT release assays to block NET, 100 nM citalopram was added to DAT and NET assays to block SERT. Synaptosomes were preloaded with radiolabeled substrate in Krebs-phosphate buffer for 1 h to reach steady state. Release assays were initiated by adding 850 μl of preloaded synaptosomes to 150 μl of test drug. Release was terminated by vacuum filtration and retained radioactivity was quantified by liquid scintillation counting. Effects of test drugs on release are expressed as percent maximum release, with maximal release (*i.e.*, 100% Emax) defined as the release produced by tyramine at doses that evoke the efflux of all “releasable” tritium by synaptosomes (*i.e.*, 10 μM tyramine for rDAT and rNET assay conditions, and 100 μM tyramine for rSERT assay conditions).

### Data analysis for synaptosome assays

Effects of test drugs on release and uptake inhibition were analyzed by nonlinear regression using GraphPad Prism 9 (GraphPad Scientific, San Diego, CA, USA). Concentration–response values for the uptake inhibition and release were fit to the equation, $Y\left(x\right)=Y_{\min}+\frac{Y_{\max}-Y_{\min}}{1+10^{\left(\left(\text{LogP}50-\text{LogX}\right)\cdot n\right)}}$, where x is the concentration of the compound tested, Y(x) is the response measured, Y_max_ is the maximal response, P_50_ is either IC_50_ (the concentration that yields half-maximal uptake inhibition response) or EC_50_ (the concentration that yields half-maximal release response), and n is the Hill slope parameter. Compounds displaying <30% of E_max_ releasing efficacy were considered inactive in the release assay, since uptake inhibitors can evoke this degree of partial release.

## Data availability

Data will be made available on request by contacting the corresponding author Harald H. Sitte (harald.sitte@meduiniwien.ac.at).

## Conflict of interest

The authors declare that they have no conflicts of interest with the contents of this article.
