# Supplementary material for: Prolintane analogs as hybrid monoamine transporter ligands: Structural determinants and species differences
Source: J Biol Chem. 2025 Nov 4;301(12):110903. doi: 10.1016/j.jbc.2025.110903 (PMC12720098; doi:10.1016/j.jbc.2025.110903)
Supplement: Supporting information [file mmc1.docx]

**Supporting Information**

**Title**

Prolintane analogs as hybrid monoamine transporter ligands: structural determinants and species differences.

**Authors**
Nina Kastner^1^, Mohammad N. Islam^1^, Michael Dybek^2^, Evelyn Roth^2^, Simon Heisinger^1^, Marion Holy^1^, Kathrin Jäntsch^1^, Donna Walther^3^, Thomas Stockner^1^, Michael H. Baumann^3^, Simon D. Brandt^4^, Jason Wallach^2^, Harald H. Sitte^1,5,6^, Oliver Kudlacek^1^

**Affiliations**

^1^ Center for Physiology and Pharmacology, Institute of Pharmacology, Medical University of Vienna, 1090 Vienna, Austria

^2^ Department of Pharmaceutical Sciences, Philadelphia College of Pharmacy, Saint Joseph’s University, Philadelphia, PA 19104, USA

^3^ Designer Drug Research Unit, National Institute on Drug Abuse Intramural Research Program, Baltimore

^4^ The Alexander Shulgin Research Institute, Lafayette, CA 94549, United States.

^5^ Hourani Center for Applied Scientific Research, Al-Ahliyya Amman University, 19328 Amman, Jordan

^6^ Center for Addiction Research and Science, Medical University of Vienna, 1090 Vienna, Austria

**Corresponding author**

Harald H. Sitte (harald.sitte@meduniwien.ac.at)

**Short/running title**

Monoamine transporter activity of prolintane analogs

**Keywords**

Prolintane analogs, monoamine transporters, structure-activity relationship, serotonin transporter mediated efflux, molecular docking approach

1. ***Supporting Methods***

*Preparation of 1-(1-phenylpentan-2-yl)pyrrolidine (Prolintane)*

A heat-dried 100 mL round bottom flask (RBF), teflon-coated magnetic stir bar were flushed with argon gas while heating externally. The system was allowed to cool down back to room temperature and then 40 mL of dry (3 Å molecular sieves) tetrahydrofuran (THF) was added to the RBF, followed by zinc dust (<10 μm, 1.81 g, 27.8 mmol), and trifluoroacetic acid (269 μL, 3.5 mmol). This was allowed to react for a few minutes and then benzyl bromide (4.76 g, 3.31 mL, 27.8 mmol) was added. The resulting exothermic reaction was stirred for 15 min at which point it had recovered to room temperature (RT). Then pyrrolidine (1.09 g, 1.26 mL, 15.3 mmol) and butyraldehyde (1.00 g, 1.25 mL, 13.9 mmol) were added in rapid succession with a pipettor and the reaction stirred overnight. Product formation was confirmed using TLC, mobile phase: ~1:4 EtOAc:hexanes + 1% TEA. The reaction was then quenched by pouring into 200 mL of 1 M HCl solution which was washed with EtOAc (2 x 75 mL). These organic washes were pooled and then extracted with 1 M HCl (3 x 75 mL). All acidic aqueous phases were combined with the original acidic phase, and were then made basic with 28% NH_4_OH to pH ~10 and then extracted with EtOAc (3 x 100 mL). The organic phases were pooled, washed with saline (~30 mL), dried over anhydrous Na_2_SO_4_ and the solvent removed *in vacuo* to yield 3.4 g of the crude freebase as a viscous yellow oil. This material was taken and converted into the HCl salt by first dissolving in 20 mL of 200 proof EtOH and titrating, while vigorously mixing, with a slight molar excess of concentrated HCl solution. The solvent was then evaporated under a stream of warm air. Additional ethanol was added and this cycle repeated three times driving off excess HCl and moisture to ultimately yielding an off-white crystalline solid. Subsequent syntheses on this series found that exposure to high vac to remove as much of residual solvent as possible helped with solid salt formation. These solids were washed with Et_2_O (3 x 5 mL), dried with gentle heating, and then dissolved in a small amount (~5 mL) of 200 proof EtOH. 40 mL of Et_2_O was then added and the solution was stored in a -20 °C freezer overnight. The resulting crystalline solids were collected by gravity filtration and the solids were washed with Et_2_O (2 x 10 mL), air dried, and then recrystallized an additional 3 times. The resulting white crystalline solids were dried with gentle heating followed by vacuum to yield prolintane HCl (3.19 g, 12.6 mmol, 90.6% yield) as a white crystalline powder (m.p. 131.4-132.7 °C). HRMS (ASAP): [M+H]^+^ theoretical for C_15_H_24_N^+^ m/z 218.1903, observed m/z 218.1896, Δppm = -3.2 ppm. [HCl] ^1^H NMR (400 MHz, DMSO) δ 11.08 (s, 1NH^+^), 7.38-7.28 (m, 4H), 7.28-7.21 (m, 1H), 3.60-3.41 (m, 3H), 3.24 (dd, *J* = 13.7, 4.0 Hz, 1H), 3.17-3.02 (m, 2H), 2.85 (dd, *J* = 13.6, 9.9 Hz, 1H), 2.05-1.82 (m, 4H), 1.64-1.49 (m, 2H), 1.36-1.23 (m, 1H), 1.23-1.11 (m, 1H), 0.72 (t, *J* = 7.3 Hz, 3H).^13^C NMR (101 MHz, DMSO) δ 137.26 (1C), 129.06 (2C), 128.55 (2C), 126.72 (1C), 64.13 (1C), 50.91 (1C), 50.66 (1C), 35.65 (1C), 31.42 (1C), 23.00 (1C), 22.95 (1C), 18.15 (1C), 13.74 (1C). HPLC Purity (7:3 Buffer:ACN, 220 nm): 100.0%, Retention Time (RT): 4.95 min.

*Preparation of 1-[1-(2-methylphenyl)pentan-2-yl]pyrrolidine (2-Me-Prolintane)*

2-Methyl-prolintane was prepared as described above using butyraldehyde (0.82 g, 1.03 mL, 11.4 mmol), pyrrolidine (0.89 g, 1.03 mL, 12.5 mmol), 2-methylbenzyl bromide (4.22 g, 3.06 mL, 22.8 mmol), zinc dust (1.49 g, 22.8 mmol) and TFA (220 μL, 2.85 mmol) in 40 mL of THF as the starting materials. This crude freebase material (amber oil) was purified utilizing flash column chromatography with silica gel and a mobile phase consisting hexanes:EtOAc (4:1) containing 1% triethylamine (TEA) to provide 2-Me-prolintane (2.28 g, 9.85 mmol, 86.4% yield) as a transparent oil. The HCl salt was prepared as described for prolintane HCl and obtained as orange tinted crystalline solids (m.p. 125.9-127.4 °C). HRMS (ASAP): [M+H]^+^ theoretical for C_16_H_26_N^+^ m/z 232.2060, observed m/z 232.2052, Δppm = -3.4 ppm. [HCl] ^1^H NMR (400 MHz, DMSO) δ 10.85 (s, 1NH^+^), 7.27-7.20 (m, 1H), 7.20-7.10 (m, 3H), 3.67-3.47 (m, 3H), 3.24 (dd, *J* = 13.7, 4.1 Hz, 1H), 3.17-2.99 (m, 2H), 2.87 (dd, *J* = 13.7, 10.5 Hz, 1H), 2.32 (s, 1CH_3_), 2.04-1.82 (m, 4H), 1.65-1.45 (m, 2H), 1.34-1.14 (m, 1H), 1.14-0.96 (m, 1H), 0.71 (t, *J* = 7.3 Hz, 3H). ^13^C NMR (101 MHz, DMSO) δ 136.32 (1C), 135.20 (1C), 130.52 (1C), 130.03 (1C), 127.01 (1C), 126.07 (1C), 62.65 (s, 1C), 51.09 (1C), 50.54 (1C), 33.51 (1C), 31.40 (1C), 22.98 (1C), 22.96 (1C), 19.36 (s, 1C), 18.42 (1C), 13.83 (1C). HPLC Purity (7:3 Buffer:ACN, 220 nm): 100.0%, RT: 7.19 min.

*Preparation of 1-[1-(3-methylphenyl)pentan-2-yl]pyrrolidine (3-Me-Prolintane)*

3-Me-prolintane was prepared as described for prolintane except using butyraldehyde (0.82 g, 1.03 mL, 11.4 mmol), pyrrolidine (0.89 g, 1.03 mL, 12.5 mmol), 3-methylbenzyl bromide (4.22 g, 3.08 mL, 22.8 mmol), zinc dust (1.49 g, 22.8 mmol) and TFA (220 μL, 2.85 mmol) in 40 mL of THF. The resulting crude freebase material (amber oil) was purified utilizing flash column chromatography on silica gel and eluted with a mixture of hexanes:EtOAc (4:1) containing 1% TEA to provide 3-Me-prolintane (1.94 g, 8.38 mmol, 73.5% yield) as a colorless transparent oil. The HCl salt was prepared as described for prolintane HCl and obtained as orange tinted crystalline solids (m.p. 114.6-116.2 °C). HRMS (ASAP): [M+H]^+^ theoretical for C_16_H_26_N^+^ m/z 232.2060, observed m/z 232.2050, Δppm = -4.3 ppm. . [HCl] ^1^H NMR (400 MHz, DMSO) δ 10.67 (s, 1NH^+^), 7.21 (t, *J* = 7.5 Hz, 1H), 7.11 (s, 1H), 7.07 (t, *J* = 7.0 Hz, 2H), 3.64-3.47 (m, 3H)*, 3.17 (dd, *J* = 13.7, 4.1 Hz, 1H), 3.16-3.00 (m, 2H), 2.79 (dd, *J* = 13.7, 9.9 Hz, 1H), 2.28 (s, 1CH_3_), 2.03-1.81 (m, 4H), 1.62-1.47 (m, 2H), 1.35-1.23 (m, 1H), 1.23-1.11 (m, 1H), 0.73 (t, *J* = 7.3 Hz, 3H). * = Coalescing with H_2_O. ^13^C NMR (101 MHz, DMSO) δ 137.80 (1C), 137.17 (1C), 129.75 (1C), 128.57 (1C), 127.51 (1C), 126.25 (1C), 64.33 (1C), 51.18 (1C), 50.94 (1C), 35.59 (1C), 31.46 (1C), 23.05 (1C), 23.03 (1C), 21.07 (1C), 18.10 (1C), 13.87 (1C). HPLC Purity (7:3 Buffer:ACN, 220 nm): 100.0%, RT: 8.53 min.

*Preparation of 1-[1-(4-methylphenyl)pentan-2-yl]pyrrolidine (4-Me-Prolintane)*

4-Me-Prolintane was prepared as described above using butyraldehyde (0.82 g, 1.03 mL, 11.4 mmol), pyrrolidine (0.89 g, 1.03 mL, 12.5 mmol), 4-methylbenzyl bromide (4.22 g, 22.8 mmol), zinc dust (1.49 g, 22.8 mmol) and TFA (220 μL, 2.85 mmol) in 40 mL of THF as the starting materials. This crude freebase material (amber oil) was purified utilizing column chromatography with silica gel and eluted with a mixture of hexanes and EtOAc (4:1) containing 1% TEA to give a transparent oil as the purified product, 4-Me-prolintane (2.32 g, 10.0 mmol, 87.7% yield). The HCl salt was prepared as described for prolintane and collected as an orange-tinted crystalline solid (m.p. 118-120.2 °C). HRMS (ASAP): [M+H]^+^ theoretical for C_16_H_26_N^+^ m/z 232.2060, observed m/z 232.2050, Δppm = - 4.3 ppm. [HCl] ^1^H NMR (400 MHz, DMSO) δ 10.60 (s, 1NH^+^), 7.18 (dm, *J* = 8.1 Hz, 2H), 7.14 (dm, *J* = 8.0 Hz, 2H), 3.62-3.46 (m, 3H)*, 3.15 (dd, *J* = 13.8, 4.1 Hz, 1H), 3.12-3.00 (m, 2H), 2.78 (dd, *J* = 13.7, 9.9 Hz, 1H), 2.27 (s, 1CH_3_), 2.04-1.81 (m, 4H), 1.61-1.44 (m, 2H), 1.36-1.22 (m, 1H), 1.22-1.07 (m, 1H), 0.73 (t, *J* = 7.3 Hz, 3H).* = Coalescing with H_2_O. ^13^C NMR (101 MHz, DMSO) δ 135.88 (1C), 134.08 (1C), 129.27 (2C), 129.06 (2C), 64.39 (1C), 51.16 (1C), 50.94 (1C), 35.23 (1C), 31.39 (1C), 23.05 (1C), 23.02 (1C), 20.73 (1C), 18.11 (1C), 13.89 (1C). HPLC Purity (7:3 Buffer:ACN, 220 nm): 100.0%, RT: 8.41 min.

*Preparation of 1-[1-(2-fluorophenyl)pentan-2-yl]pyrrolidine (2-F-Prolintane)*

2-F-Prolintane was prepared as described above using butyraldehyde (0.82 g, 1.03 mL, 11.4 mmol), pyrrolidine (0.89 g, 1.03 mL, 12.5 mmol), 2-fluorobenzyl bromide (4.31 g, 2.75 mL, 22.8 mmol), zinc dust (1.49 g, 22.8 mmol) and TFA (220 μL, 2.85 mmol) in 40 mL of THF as the starting materials. This crude freebase material (amber oil) was purified utilizing column chromatography with silica gel and eluted with a mixture of hexanes and EtOAc (4:1) containing 1% TEA to give an off clear oil as the pure product, 2-F-prolintane (1.79 g, 7.61 mmol, 66.8% yield). The HCl salt was prepared as described for prolintane HCl and obtained as white crystalline solids (m.p. 103.2-105.9 °C). HRMS (ASAP): [M+H]^+^ theoretical for C_15_H_23_FN^+^ m/z 236.1809, observed m/z 236.1799, Δppm = - 4.2 ppm. [HCl] ^1^H NMR (400 MHz, DMSO) δ 10.79 (s, 1NH^+^), 7.44 (td, *J* = 7.7, 1.5 Hz, 1H), 7.39-7.29 (m, 1H), 7.27-7.14 (m, 2H), 3.65-3.54 (m, 1H), 3.54-3.47 (m, 2H)*, 3.20 (dd, *J* = 13.8, 3.9 Hz, 1H), 3.17-3.02 (m, 2H), 2.97 (dd, *J* = 13.8, 10.0 Hz, 1H), 2.05-1.83 (m, 4H), 1.64-1.48 (m, 2H), 1.37-1.24 (m, 1H), 1.24-1.13 (m, 1H), 0.73 (t, *J* = 7.3 Hz, 3H). * = Coalescing with H_2_O. ^13^C NMR (101 MHz, DMSO) δ 160.66 (d, *J* = 243.2 Hz, 1C), 131.80 (d, *J* = 4.1 Hz, 1C), 129.34 (d, *J* = 8.6 Hz, 1C), 124.82 (d, *J* = 3.0 Hz, 1C), 123.90 (d, *J* = 15.3 Hz, 1C), 115.54 (d, *J* = 21.6 Hz, 1C), 63.27 (d, *J* = 1.3 Hz, 1C) 51.27 (1C), 50.96 (1C), 31.66 (1C), 29.28 (d, *J* = 1.6 Hz, 1C), 23.02 (2C), 18.10 (1C), 13.84 (1C). HPLC Purity (7:3 Buffer:ACN, 220 nm): 100.0%, RT: 5.39 min.

*Preparation of 1-[1-(3-fluorophenyl)pentan-2-yl]pyrrolidine (3-F-Prolintane)*

3-F-Prolintane was prepared as described above using butyraldehyde (0.50 g, 0.63 mL, 6.93 mmol), pyrrolidine (0.54 g, 0.63 mL, 7.62 mmol), 3-fluorobenzyl bromide (2.63 g, 1.71 mL, 13.9 mmol), zinc dust (0.91 g, 13.9 mmol) and TFA (133 μL, 1.73 mmol) in 20 mL of THF as the starting materials. This crude freebase material (amber oil) was purified utilizing column chromatography with silica gel and eluted with a mixture of hexanes and EtOAc (4:1) containing 1% TEA to give 3-F-prolintane (0.30 g, 1.27 mmol, 18.3% yield) as a transparent oil. The HCl salt was prepared as described for prolintane HCl and obtained as white crystalline solids (m.p. 123.6-126.6 °C). HRMS (ASAP): [M+H]^+^ theoretical for C_15_H_23_FN^+^ m/z 236.1809, observed m/z 236.1801, Δppm = - 3.4 ppm. [HCl] ^1^H NMR (400 MHz, DMSO) δ 10.71 (s, 1NH^+^), 7.38 (td, *J* = 7.9, 6.3 Hz, 1H), 7.21 (dt, *J* = 10.4, 1.9 Hz, 1H), 7.15 (d, *J* = 7.7 Hz, 1H), 7.10 (td, *J* = 12.8, 2.5 Hz, 1H), 3.68-3.48 (m, 3H)*, 3.23 (dd, *J* = 13.7, 4.0 Hz, 1H), 3.18-3.02 (m, 2H), 2.88 (dd, *J* = 13.7, 9.9 Hz, 1H), 2.01-1.85 (m, 4H), 1.61-1.49 (m, 2H), 1.34-1.22 (m, 1H), 1.22-1.12 (m, 1H), 0.73 (t, *J* = 7.3 Hz, 3H). * = Coalescing with H_2_O. [HCl] ^13^C NMR (101 MHz, DMSO) δ 162.32 (d, *J* = 243.8 Hz, 1C), 140.22 (d, *J* = 7.7 Hz, 1C), 130.61 (d, *J* = 8.7 Hz, 1C), 125.44 (d, *J* = 2.3 Hz, 1C), 115.95 (d, *J* = 21.3 Hz, 1C), 113.75 (d, *J* = 20.7 Hz, 1C), 63.94 (1C), 51.13 (1C), 50.93 (1C), 35.33 (d, *J* = 1.5 Hz. 1C), 31.49 (1C), 23.10 (1C), 23.05 (1C), 18.15 (1C), 13.86 (s, 1C). HPLC Purity (7:3 Buffer:ACN, 220 nm): 100.0%, RT: 5.71 min.

*Preparation of 1-[1-(4-fluorophenyl)pentan-2-yl]pyrrolidine (4-F-Prolintane)*

4-F-Prolintane was prepared as described above using butyraldehyde (0.82 g, 1.03 mL, 11.4 mmol), pyrrolidine (0.89 g, 1.03 mL, 12.5 mmol), 4-fluorobenzyl bromide (4.31 g, 2.84 mL, 22.8 mmol), zinc dust (1.49 g, 22.8 mmol) and TFA (220 μL, 2.85 mmol) in 40 mL of THF as the starting materials. This crude freebase material (amber oil) was purified utilizing column chromatography with silica gel and eluted with a mixture of hexanes and EtOAc (4:1) containing 1% TEA to give 4-F-prolintane (1.31 g, 5.56 mmol, 48.8% yield) as a transparent oil. The HCl salt was prepared as described for prolintane HCl and obtained as a white crystalline solid (m.p. 109.4-111.0 °C). HRMS (ASAP): [M+H]^+^ theoretical for C_15_H_23_FN^+^ m/z 236.1809, observed m/z 236.1802, Δppm = - 3.0 ppm. [HCl] ^1^H NMR (400 MHz, DMSO) δ 10.69 (s, 1NH^+^), 7.36 (dd, *J* = 8.2, 5.7 Hz, 2H), 7.17 (at, *J* = 8.8 Hz, 2H), 3.62-3.47 (m, 3H)*, 3.20 (dd, *J* =13.7, 3.8 Hz, 1H), 3.16-3.01 (m, 2H), 2.85 (dd, *J* =13.6, 10.0 Hz, 1H), 2.07-1.79 (m, 4H), 1.61-1.46 (m, 2H), 1.35-1.21 (m, 1H), 1.21-1.08 (m, 1H), 0.73 (t, *J* = 7.2 Hz, 3H). * = Coalescing with H_2_O. [HCl] ^13^C NMR (101 MHz, DMSO) δ 161.18 (d, *J* =242.3 Hz, 1C), 133.39 (d, *J* = 3.4 Hz, 1C), 131.12 (d, *J* = 8.1 Hz, 2C), 115.44 (d, *J* = 21.2 Hz, 2C), 64.23 (1C), 51.15 (1C), 50.87 (1C), 34.81 (1C), 31.30 (1C), 23.09 (1C), 23.05 (1C), 18.20 (1C), 13.87 (s, 1C). HPLC Purity (7:3 Buffer:ACN, 220 nm): 97.2%, RT: 5.96 min.

1. ***NMR Assignments***


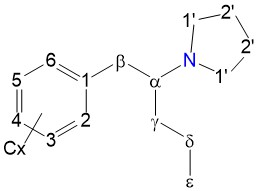


**Figure S1:** Prolintane Scaffold Numbering Scheme for NMR Assignments

**Table S1:** ^1^H NMR Assignments

| **Proton** | **Prolintane** | **2-F-Prolintane** | **3-F-Prolintane** | **4-F-Prolintane** | **2-Me-Prolintane** | **3-Me-Prolintane** | **4-Me-Prolintane** |
| --- | --- | --- | --- | --- | --- | --- | --- |
| H_α_ | 3.60-3.41 (m, 1H)  Coalescing with H_1’_ | 3.64-3.55 (m, 1H) | 3.68-3.48 (m, 1H)  Coalescing with H_1’_ and H_2_O | 3.62-3.47 (m, 1H)  Coalescing with H_1’_ and H_2_O | 3.67-3.47 (m, 1H)  Coalescing with H_1’_ | 3.64-3.47 (m, 1H)  Coalescing with H_1’_ and H_2_O | 3.62-3.46 (m, 1H)  Coalescing with H_1’_and H_2_O |
| H_β_ | 3.24 (dd, *J* = 13.7, 4.0 Hz, 1H)  2.85 (dd, *J* = 13.6, 9.9 Hz, 1H) | 3.20 (dd, *J* = 13.8, 3.9 Hz, 1H)  2.97 (dd, *J* = 13.8, 10.0 Hz, 1H) | 3.23 (dd, *J* = 13.7, 4.0 Hz, 1H)  2.88 (dd, *J* = 13.7, 9.9 Hz, 1H) | 3.20 (dd, *J* = 13.7, 3.8 Hz, 1H)  2.85 (dd, *J* = 13.6, 10 Hz, 1H) | 3.24 (dd, *J* = 13.7, 4.1 Hz, 1H)  2.87 (dd, *J* = 13.7, 10.5 Hz, 1H) | 3.17 (dd, *J* = 13.7, 4.1 Hz, 1H)  2.79 (dd, *J* = 13.7, 9.9 Hz, 1H) | 3.15 (dd, *J* = 13.8, 4.1 Hz, 1H)  2.78 (dd, *J* = 13.7 Hz, 9.9 Hz, 1H) |
| H_γ_ | 1.64-1.49 (m, 2H) | 1.64-1.48 (m, 2H) | 1.61-1.49 (m, 2H) | 1.61-1.46 (m, 2H) | 1.65-1.45 (m, 2H) | 1.62-1.47 (m, 2H) | 1.61-1.44 (m, 2H) |
| H_δ_ | 1.36-1.23 (m, 1H)  1.23-1.11 (m, 1H) | 1.37-1.24 (m, 1H)  1.24-1.13 (m, 1H) | 1.34-1.22 (m, 1H)  1.22-1.12 (m, 1H) | 1.35-1.21 (m, 1H)  1.21-1.08 (m, 1H) | 1.34-1.14 (m, 1H)  1.14-0.99 (m, 1H) | 1.35-1.23 (m, 1H)  1.23-1.11 (m, 1H) | 1.36-1.22 (m, 1H)  1.22-1.07 (m, 1H) |
| H_ε_ | 0.72 (t, *J* = 7.3 Hz, 3H) | 0.73 (t, *J* = 7.3 Hz, 3H) | 0.73 (t, *J* = 7.3 Hz, 3H) | 0.73 (t, *J* = 7.2 Hz, 3H) | 0.71 (t, *J* = 7.3 Hz, 3H) | 0.73 (t, *J* = 7.3 Hz, 3H) | 0.73 (t, *J* = 7.3 Hz, 3H) |
| H_2_ | 7.38-7.28 (m, 1H)  Coalescing with H_3,5,6_ | - | 7.21 (dt, *J* = 10.4, 1.9 Hz, 1H) | 7.36 (dd, *J =* 8.2, 5.7 Hz, 2H)  Coalescing with H_6_ | - | 7.11 (s, 1H) | 7.18 (dm, *J* = 8.1 Hz, 1H) |
| H_3_ | 7.38-7.28 (m, 1H)  Coalescing with H_2,5,6_ | 7.27-7.14 (m, 1H)  Coalescing with H_5_ | - | 7.17 (at, *J* = 8.8 Hz, 1H)  Coalescing with H_5_ | 7.27-7.20 (m, 1H) | - | 7.14 (dm, *J* = 8.0 Hz, 1H) |
| H_4_ | 7.28-7.21 (m, 1H) | 7.39-7.29 (m, 1H) | 7.10 (td, *J* = 12.8, 2.5 Hz, 1H) | - | 7.20-7.10 (m, 1H)  Coalescing with H_5,6_ | 7.07 (t, *J* = 7.0 Hz, 1H)  Coalescing with H_6_ | - |
| H_5_ | 7.38-7.28 (m, 1H)  Coalescing with H_2,3,6_ | 7.27-7.14 (m, 1H)  Coalescing with H_3_ | 7.38 (td, *J* = 7.9, 6.3 Hz, 1H) | 7.17 (at, *J* = 8.8 Hz, 1H)  Coalescing with H_3_ | 7.20-7.10 (m, 1H)  Coalescing with H_4,6_ | 7.21 (t, *J* = 7.5 Hz, 1H) | 7.14 (dm, *J* = 8.0 Hz, 1H) |
| H_6_ | 7.38-7.28 (m, 1H)  Coalescing with H_2,3,5_ | 7.44 (td, *J* = 7.7, 1.5 Hz, 1H) | 7.15 (d, *J* = 7.7 Hz, 1H) | 7.36 (dd, *J =* 8.2, 5.7 Hz, 2H)  Coalescing with H_2_ | 7.20-7.10 (m, 1H)  Coalescing with H_4,5_ | 7.07 (t, *J* = 7.0 Hz, 1H)  Coalescing with H_4_ | 7.18 (dm, *J* = 8.1 Hz, 1H) |
| H_1’_ | 3.60-3.41 (m, 2H)  Coalescing with H_α_,  3.17-3.02 (m, 2H) | 3.55-3.47 (m, 2H)  Coalescing with H_2_O  3.17-3.02 (m, 2H) | 3.64-3.48 (m, 2H)  Coalescing with H_α_, and H_2_O  3.18-3.02 (m, 2H) | 3.61-3.48 (m, 2H)  Coalescing with H_α_, and H_2_O  3.16-3.01 (m, 2H) | 3.67-3.47 (m, 2H)  Coalescing with H_α_  3.17-2.99 (m, 2H) | 3.64-3.47 (m, 2H)  Coalescing with H_α_ and H_2_O  3.16-3.00 (m, 2H) | 3.62-3.46 (m, 2H)  Coalescing with H_α_ and H_2_O  3.12-3.00 (m, 2H) |
| H_2’_ | 2.05-1.82 (m, 4H) | 2.05-1.83 (m, 4H) | 2.01-1.85 (m, 4H) | 2.07-1.79 (m, 4H) | 2.04-1.82 (m, 4H) | 2.03-1.81 (m, 4H) | 2.04-1.81 (m, 4H) |
| NH^+^ | 11.08 (s, 1NH^+^) | 10.79 (s, 1NH^+^) | 10.71 (s, 1NH^+^) | 10.69 (s, 1NH^+^) | 10.85 (s, 1NH^+^) | 10.67 (s, 1NH^+^) | 10.60 (s, 1NH^+^) |
| C_x_ | - | - | - | - | 2.32 (s, 1CH_3_) | 2.28 (s, 1CH_3_) | 2.27 (s, 1CH_3_) |

**Table S2:** ^13^C NMR Assignments

| **Carbon** | **Prolintane** | **2-F-Prolintane** | **3-F-Prolintane** | **4-F-Prolintane** | **2-Me-Prolintane** | **3-Me-Prolintane** | **4-Me-Prolintane** |
| --- | --- | --- | --- | --- | --- | --- | --- |
| C_α_ | 64.13 | 63.27 (d, *J* = 1.3 Hz) | 63.94 | 64.23 | 62.65 | 64.33 | 64.39 |
| C_β_ | 35.65 | 29.28 (d, *J* = 1.6 Hz) | 35.33 (d, *J* = 1.5 Hz) | 34.81 | 33.51 | 35.59 | 35.23 |
| C_γ_ | 31.42 | 31.66 | 31.49 | 31.30 | 31.40 | 31.46 | 31.39 |
| C_δ_ | 18.15 | 18.10 | 18.15 | 18.20 | 18.42 | 18.10 | 18.11 |
| C_ε_ | 13.74 | 13.84 | 13.86 | 13.87 | 13.91 | 13.87 | 13.89 |
| C_1_ | 137.26 | 123.90 (d, *J* = 15.3 Hz) | 140.22 (d, *J* = 7.7 Hz) | 133.39 (d, *J* = 3.4 Hz) | 135.20 | 137.17 | 134.08 |
| C_2_ | 129.06 | 160.66 (d, *J* = 243.2 Hz) | 115.95 (d, *J* = 21.3 Hz) | 131.12 (d, *J* = 8.1 Hz) | 136.32 | 129.75 | 129.06 |
| C_3_ | 128.55 | 115.54 (d, *J* = 21.6 Hz) | 162.32 (d, *J* = 243.8 Hz) | 115.44 (d, *J* = 21.2 Hz) | 130.52 | 137.80 | 129.27 |
| C_4_ | 126.72 | 129.34 (d, *J* = 8.6 Hz) | 113.75 (d, *J* = 20.7 Hz) | 161.18 (d, *J* = 242.3 Hz) | 127.01 | 127.51 | 135.88 |
| C_5_ | 128.55 | 124.82 (d, *J* = 3.0 Hz) | 130.61 (d, *J* = 8.7 Hz) | 115.44 (d, *J* = 21.2 Hz) | 126.07 | 128.57 | 129.27 |
| C_6_ | 129.06 | 131.80 (d, *J* = 4.1 Hz) | 125.44 (d, *J* = 2.3 Hz) | 131.12 (d, *J* = 8.1 Hz) | 130.03 | 126.25 | 129.06 |
| C_1’_ | 50.91  50.66 | 51.27  50.96 | 51.13  50.93 | 51.15  50.87 | 51.09  50.54 | 51.18  50.94 | 51.16  50.94 |
| C_2’_ | 23.00  22.95 | 23.02 | 23.10  23.05 | 23.09  23.05 | 22.98  22.96 | 23.05  23.03 | 23.05  23.02 |
| C_x_ | - | - | - | - | 19.36 | 21.07 | 20.73 |

1. ***Supporting Results***
   1. ***Uptake Inhibition Statistics: log transformed IC_50_ values***

**Table S3:** Summary of one-way ANOVA of hSERT uptake inhibition.

| ANOVA table | SS | DF | MS | F (DFn, DFd) | P value |
| --- | --- | --- | --- | --- | --- |
| Treatment (between columns) | 5.223 | 6 | 0.8705 | F (6, 18) = 52.96 | P<0.0001 |
| Residual (within columns) | 0.2959 | 18 | 0.01644 |  |  |
| Total | 5.519 | 24 |  |  |  |

**Table S4:** Summary of Tukey's multiple comparisons test of hSERT uptake inhibition.

| Šídák's multiple  comparisons test | Mean diff, | 95,00% CI  of diff, | Below threshold? | Summary | Adjusted  P Value |
| --- | --- | --- | --- | --- | --- |
| Prolintane vs. 2-Methylprolintane | 0.8652 | 0.5656 to 1.165 | Yes | **** | <0.0001 |
| Prolintane vs. 3-Methylprolintane | 0.99 | 0.6665 to 1.314 | Yes | **** | <0.0001 |
| Prolintane vs. 4-Methylprolintane | 1.053 | 0.7531 to 1.352 | Yes | **** | <0.0001 |
| Prolintane vs. 2-Fluoroprolintane | -0.0782 | -0.3778 to 0.2214 | No | ns | 0.9738 |
| Prolintane vs. 3-Fluoroprolintane | 0.3249 | 0.001283 to 0.6484 | Yes | * | 0.0487 |
| Prolintane vs. 4-Fluoroprolintane | 0.1645 | -0.1591 to 0.4881 | No | ns | 0.6366 |
| 2-Methylprolintane  vs. 3-Methylprolintane | 0.1249 | -0.1987 to 0.4484 | No | ns | 0.8544 |
| 2-Methylprolintane  vs. 4-Methylprolintane | 0.1875 | -0.1121 to 0.4871 | No | ns | 0.4099 |
| 2-Methylprolintane  vs. 2-Fluoroprolintane | -0.9434 | -1.243 to -0.6438 | Yes | **** | <0.0001 |
| 2-Methylprolintane  vs. 3-Fluoroprolintane | -0.5403 | -0.8639 to -0.2168 | Yes | *** | 0.0005 |
| 2-Methylprolintane  vs. 4-Fluoroprolintane | -0.7007 | -1.024 to -0,.771 | Yes | **** | <0.0001 |
| 3-Methylprolintane vs. 4-Methylprolintane | 0.06264 | -0.2609 to 0,3862 | No | ns | 0.9943 |
| 3-Methylprolintane vs. 2-Fluoroprolintane | -1.068 | -1,392 to -0.7447 | Yes | **** | <0.0001 |
| 3-Methylprolintane vs. 3-Fluoroprolintane | -0.6652 | -1.011 to -0.3193 | Yes | **** | <0.0001 |
| 3-Methylprolintane vs. 4-Fluoroprolintane | -0.8255 | -1.171 to -0.4796 | Yes | **** | <0,0001 |
| 4-Methylprolintane vs. 2-Fluoroprolintane | -1.131 | -1.430 to -0.8313 | Yes | **** | <0.0001 |
| 4-Methylprolintane vs. 3-Fluoroprolintane | -0.7278 | -1.051 to -0.4043 | Yes | **** | <0.0001 |
| 4-Methylprolintane vs. 4-Fluoroprolintane | -0.8882 | -1.212 to -0.5646 | Yes | **** | <0.0001 |
| 2-Fluoroprolintane vs. 3-Fluoroprolintane | 0.403 | 0.07948 to 0.7266 | Yes | ** | 0.0095 |
| 2-Fluoroprolintane vs. 4-Fluoroprolintane | 0.2427 | -0.08087 to 0.5663 | No | ns | 0.2241 |
| 3-Fluoroprolintane  vs. 4-Fluoroprolintane | -0.1604 | -0.5063 to 0.1856 | No | ns | 0.7235 |

**Table S5:** Summary of one-way ANOVA of hDAT uptake inhibition.

| ANOVA table | SS | DF | MS | F (DFn, DFd) | P value |
| --- | --- | --- | --- | --- | --- |
| Treatment (between columns) | 2.236 | 6 | 0.3726 | F (6, 16) = 9.909 | P=0.0001 |
| Residual (within columns) | 0.6017 | 16 | 0.03761 |  |  |
| Total | 2.838 | 22 |  |  |  |

**Table S6:** Summary of Tukey's multiple comparisons test of hDAT uptake inhibition.

| Šídák's multiple  comparisons test | Mean diff, | 95,00% CI  of diff, | Below threshold? | Summary | Adjusted  P Value |
| --- | --- | --- | --- | --- | --- |
| Prolintane vs. 2-Methylprolintane | -0.6892 | -1.186 to -0.1927 | Yes | ** | 0.004 |
| Prolintane vs. 3-Methylprolintane | -0.1841 | -0.6806 to 0.3124 | No | ns | 0.867 |
| Prolintane vs. 4-Methylprolintane | 0.3492 | -0.1473 to 0.8457 | No | ns | 0.2769 |
| Prolintane vs. 2-Fluoroprolintane | -0.4784 | -0.9749 to 0.01811 | No | ns | 0.0628 |
| Prolintane vs. 3-Fluoroprolintane | -0.2362 | -0.7327 to 0.2603 | No | ns | 0.6874 |
| Prolintane vs. 4-Fluoroprolintane | -0.4513 | -0.9110 to 0.008326 | No | ns | 0.056 |
| 2-Methylprolintane  vs. 3-Methylprolintane | 0.5051 | -0.02570 to 1.036 | No | ns | 0.0677 |
| 2-Methylprolintane  vs. 4-Methylprolintane | 1.038 | 0.5077 to 1.569 | Yes | *** | 0.0001 |
| 2-Methylprolintane  vs. 2-Fluoroprolintane | 0.2108 | -0.3199 to 0.7416 | No | ns | 0.8278 |
| 2-Methylprolintane  vs. 3-Fluoroprolintane | 0.453 | -0.07776 to 0.9838 | No | ns | 0.1222 |
| 2-Methylprolintane  vs. 4-Fluoroprolintane | 0.2379 | -0.2586 to 0.7344 | No | ns | 0.6809 |
| 3-Methylprolintane vs. 4-Methylprolintane | 0.5334 | 0.002599 to 1.064 | Yes | * | 0.0485 |
| 3-Methylprolintane vs. 2-Fluoroprolintane | -0.2942 | -0.8250 to 0.2365 | No | ns | 0.5317 |
| 3-Methylprolintane vs. 3-Fluoroprolintane | -0.05206 | -0.5828 to 0.4787 | No | ns | 0.9999 |
| 3-Methylprolintane vs. 4-Fluoroprolintane | -0.2672 | -0.7637 to 0.2293 | No | ns | 0.5637 |
| 4-Methylprolintane vs. 2-Fluoroprolintane | -0.8276 | -1.358 to -0.2968 | Yes | ** | 0.0013 |
| 4-Methylprolintane vs. 3-Fluoroprolintane | -0.5854 | -1.116 to -0.05466 | Yes | * | 0.0258 |
| 4-Methylprolintane vs. 4-Fluoroprolintane | -0.8006 | -1.297 to -0.3041 | Yes | *** | 0.0009 |
| 2-Fluoroprolintane vs. 3-Fluoroprolintane | 0.2422 | -0.2886 to 0.7729 | No | ns | 0.7247 |
| 2-Fluoroprolintane vs. 4-Fluoroprolintane | 0.02705 | -0.4694 to 0.5235 | No | ns | >0.9999 |
| 3-Fluoroprolintane  vs. 4-Fluoroprolintane | -0.2151 | -0.7116 to 0.2814 | No | ns | 0.7669 |

**Table S7:** Summary of one-way ANOVA of hNET uptake inhibition.

| ANOVA table | SS | DF | MS | F (DFn, DFd) | P value |
| --- | --- | --- | --- | --- | --- |
| Treatment (between columns) | 2.065 | 6 | 0.3441 | F (6, 24) = 18.86 | P<0.0001 |
| Residual (within columns) | 0.4378 | 24 | 0.01824 |  |  |
| Total | 2.502 | 30 |  |  |  |

**Table S8:** Summary of Tukey's multiple comparisons test of hNET uptake inhibition.

| Šídák's multiple  comparisons test | Mean diff, | 95,00% CI  of diff, | Below threshold? | Summary | Adjusted  P Value |
| --- | --- | --- | --- | --- | --- |
| Prolintane vs. 2-Methylprolintane | -0.2787 | -0.5696 to 0.01228 | No | ns | 0.0667 |
| Prolintane vs. 3-Methylprolintane | -0.3304 | -0.6214 to -0.03949 | Yes | * | 0.0189 |
| Prolintane vs. 4-Methylprolintane | -0.1311 | -0.4220 to 0.1599 | No | ns | 0.7717 |
| Prolintane vs. 2-Fluoroprolintane | -0.2293 | -0.5360 to 0.07738 | No | ns | 0.241 |
| Prolintane vs. 3-Fluoroprolintane | 0.2449 | -0.06182 to 0.5515 | No | ns | 0.1816 |
| Prolintane vs. 4-Fluoroprolintane | -0.6928 | -0.9994 to -0.3861 | Yes | **** | <0.0001 |
| 2-Methylprolintane  vs. 3-Methylprolintane | -0.05177 | -0.3261 to 0.2225 | No | ns | 0.996 |
| 2-Methylprolintane  vs. 4-Methylprolintane | 0.1476 | -0.1267 to 0.4219 | No | ns | 0.6053 |
| 2-Methylprolintane  vs. 2-Fluoroprolintane | 0.04936 | -0.2416 to 0.3403 | No | ns | 0.9978 |
| 2-Methylprolintane  vs. 3-Fluoroprolintane | 0.5235 | 0.2326 to 0.8144 | Yes | *** | 0.0001 |
| 2-Methylprolintane  vs. 4-Fluoroprolintane | -0.4141 | -0.7051 to -0.1232 | Yes | ** | 0.0021 |
| 3-Methylprolintane vs. 4-Methylprolintane | 0.1993 | -0.07495 to 0.4736 | No | ns | 0.2692 |
| 3-Methylprolintane vs. 2-Fluoroprolintane | 0.1011 | -0.1898 to 0.3921 | No | ns | 0.9169 |
| 3-Methylprolintane vs. 3-Fluoroprolintane | 0.5753 | 0.2843 to 0.8662 | Yes | **** | <0.0001 |
| 3-Methylprolintane vs. 4-Fluoroprolintane | -0.3623 | -0.6533 to -0.07141 | Yes | ** | 0.0083 |
| 4-Methylprolintane vs. 2-Fluoroprolintane | -0.09822 | -0.3892 to 0.1927 | No | ns | 0.9268 |
| 4-Methylprolintane vs. 3-Fluoroprolintane | 0.3759 | 0.08500 to 0.6669 | Yes | ** | 0.0058 |
| 4-Methylprolintane vs. 4-Fluoroprolintane | -0.5617 | -0.8526 to -0.2708 | Yes | **** | <0.0001 |
| 2-Fluoroprolintane vs. 3-Fluoroprolintane | 0.4742 | 0.1675 to 0.7808 | Yes | *** | 0.0008 |
| 2-Fluoroprolintane vs. 4-Fluoroprolintane | -0.4635 | -0.7701 to -0.1568 | Yes | ** | 0.001 |
| 3-Fluoroprolintane  vs. 4-Fluoroprolintane | -0.9376 | -1.244 to -0.6310 | Yes | **** | <0.0001 |

**Table S9:** Summary of one-way ANOVA of rSERT uptake inhibition.

| ANOVA table | SS | DF | MS | F (DFn, DFd) | P value |
| --- | --- | --- | --- | --- | --- |
| Treatment (between columns) | 5.933 | 6 | 0.9888 | F (6, 14) = 268.2 | P<0.0001 |
| Residual (within columns) | 0.05162 | 14 | 0.003687 |  |  |
| Total | 5.985 | 20 |  |  |  |

**Table S10:** Summary of Tukey's multiple comparisons test of rSERT uptake inhibition.

| Šídák's multiple  comparisons test | Mean diff, | 95,00% CI  of diff, | Below threshold? | Summary | Adjusted  P Value |
| --- | --- | --- | --- | --- | --- |
| Prolintane vs. 2-Methylprolintane | 0.8566 | 0.6873 to 1.026 | Yes | **** | <0.0001 |
| Prolintane vs. 3-Methylprolintane | 1.223 | 1.054 to 1.393 | Yes | **** | <0.0001 |
| Prolintane vs. 4-Methylprolintane | 1.506 | 1.336 to 1.675 | Yes | **** | <0.0001 |
| Prolintane vs. 2-Fluoroprolintane | 0.017 | -0.1523 to 0.1863 | No | ns | 0.9998 |
| Prolintane vs. 3-Fluoroprolintane | 0.6488 | 0.4795 to 0.8181 | Yes | **** | <0.0001 |
| Prolintane vs. 4-Fluoroprolintane | 0.419 | 0.2498 to 0.5883 | Yes | **** | <0.0001 |
| 2-Methylprolintane  vs. 3-Methylprolintane | 0.3666 | 0.1973 to 0.5359 | Yes | **** | <0.0001 |
| 2-Methylprolintane  vs. 4-Methylprolintane | 0.6489 | 0.4796 to 0.8182 | Yes | **** | <0.0001 |
| 2-Methylprolintane  vs. 2-Fluoroprolintane | -0.8396 | -1.009 to -0.6704 | Yes | **** | <0.0001 |
| 2-Methylprolintane  vs. 3-Fluoroprolintane | -0.2078 | -0.3771 to -0.03855 | Yes | * | 0.0122 |
| 2-Methylprolintane  vs. 4-Fluoroprolintane | -0.4376 | -0.6069 to -0.2683 | Yes | **** | <0.0001 |
| 3-Methylprolintane vs. 4-Methylprolintane | 0.2823 | 0.1130 to 0.4516 | Yes | *** | 0.0008 |
| 3-Methylprolintane vs. 2-Fluoroprolintane | -1.206 | -1.376 to -1.037 | Yes | **** | <0.0001 |
| 3-Methylprolintane vs. 3-Fluoroprolintane | -0.5744 | -0.7437 to -0.4051 | Yes | **** | <0.0001 |
| 3-Methylprolintane vs. 4-Fluoroprolintane | -0.8042 | -0.9735 to -0.6349 | Yes | **** | <0.0001 |
| 4-Methylprolintane vs. 2-Fluoroprolintane | -1.489 | -1.658 to -1.319 | Yes | **** | <0.0001 |
| 4-Methylprolintane vs. 3-Fluoroprolintane | -0.8567 | -1.026 to -0.6874 | Yes | **** | <0.0001 |
| 4-Methylprolintane vs. 4-Fluoroprolintane | -1.086 | -1.256 to -0.9172 | Yes | **** | <0.0001 |
| 2-Fluoroprolintane vs. 3-Fluoroprolintane | 0.6318 | 0.4625 to 0.8011 | Yes | **** | <0.0001 |
| 2-Fluoroprolintane vs. 4-Fluoroprolintane | 0.402 | 0.2328 to 0.5713 | Yes | **** | <0.0001 |
| 3-Fluoroprolintane  vs. 4-Fluoroprolintane | -0.2298 | -0.3990 to -0.06046 | Yes | ** | 0.0055 |

**Table S11:** Summary of one-way ANOVA of rDAT uptake inhibition.

| ANOVA table | SS | DF | MS | F (DFn, DFd) | P value |
| --- | --- | --- | --- | --- | --- |
| Treatment (between columns) | 1.978 | 6 | 0.3297 | F (6, 14) = 241.8 | P<0.0001 |
| Residual (within columns) | 0.01909 | 14 | 0.001363 |  |  |
| Total | 1.997 | 20 |  |  |  |

**Table S12:** Summary of Tukey's multiple comparisons test of rDAT uptake inhibition.

| Šídák's multiple  comparisons test | Mean diff, | 95,00% CI  of diff, | Below threshold? | Summary | Adjusted  P Value |
| --- | --- | --- | --- | --- | --- |
| Prolintane vs. 2-Methylprolintane | -0.7221 | -0.8250 to -0.6191 | Yes | **** | <0.0001 |
| Prolintane vs. 3-Methylprolintane | -0.2546 | -0.3576 to -0.1517 | Yes | **** | <0.0001 |
| Prolintane vs. 4-Methylprolintane | 0.2178 | 0.1149 to 0.3208 | Yes | **** | <0.0001 |
| Prolintane vs. 2-Fluoroprolintane | -0.278 | -0.3809 to -0.1750 | Yes | **** | <0.0001 |
| Prolintane vs. 3-Fluoroprolintane | 0.02471 | -0.07824 to 0.1277 | No | ns | 0.9787 |
| Prolintane vs. 4-Fluoroprolintane | -0.5405 | -0.6434 to -0.4375 | Yes | **** | <0.0001 |
| 2-Methylprolintane  vs. 3-Methylprolintane | 0.4675 | 0.3645 to 0.5704 | Yes | **** | <0.0001 |
| 2-Methylprolintane  vs. 4-Methylprolintane | 0.9399 | 0.8370 to 1.043 | Yes | **** | <0.0001 |
| 2-Methylprolintane  vs. 2-Fluoroprolintane | 0.4441 | 0.3412 to 0.5471 | Yes | **** | <0.0001 |
| 2-Methylprolintane  vs. 3-Fluoroprolintane | 0.7468 | 0.6438 to 0.8497 | Yes | **** | <0.0001 |
| 2-Methylprolintane  vs. 4-Fluoroprolintane | 0.1816 | 0.07867 to 0.2846 | Yes | *** | 0.0005 |
| 3-Methylprolintane vs. 4-Methylprolintane | 0.4725 | 0.3695 to 0.5754 | Yes | **** | <0.0001 |
| 3-Methylprolintane vs. 2-Fluoroprolintane | -0.02334 | -0.1263 to 0.07961 | No | ns | 0.984 |
| 3-Methylprolintane vs. 3-Fluoroprolintane | 0.2793 | 0.1764 to 0.3823 | Yes | **** | <0.0001 |
| 3-Methylprolintane vs. 4-Fluoroprolintane | -0.2858 | -0.3888 to -0.1829 | Yes | **** | <0.0001 |
| 4-Methylprolintane vs. 2-Fluoroprolintane | -0.4958 | -0.5988 to -0.3929 | Yes | **** | <0.0001 |
| 4-Methylprolintane vs. 3-Fluoroprolintane | -0.1931 | -0.2961 to -0.09019 | Yes | *** | 0.0003 |
| 4-Methylprolintane vs. 4-Fluoroprolintane | -0.7583 | -0.8612 to -0.6554 | Yes | **** | <0.0001 |
| 2-Fluoroprolintane vs. 3-Fluoroprolintane | 0.3027 | 0.1997 to 0.4056 | Yes | **** | <0.0001 |
| 2-Fluoroprolintane vs. 4-Fluoroprolintane | -0.2625 | -0.3654 to -0.1595 | Yes | **** | <0.0001 |
| 3-Fluoroprolintane  vs. 4-Fluoroprolintane | -0.5652 | -0.6681 to -0.4622 | Yes | **** | <0.0001 |

**Table S13:** Summary of one-way ANOVA of rNET uptake inhibition.

| ANOVA table | SS | DF | MS | F (DFn, DFd) | P value |
| --- | --- | --- | --- | --- | --- |
| Treatment (between columns) | 1.858 | 6 | 0.3097 | F (6, 14) = 96.56 | P<0.0001 |
| Residual (within columns) | 0.0449 | 14 | 0.003207 |  |  |
| Total | 1.903 | 20 |  |  |  |

**Table S14:** Summary of Tukey's multiple comparisons test of rNET uptake inhibition.

| Šídák's multiple  comparisons test | Mean diff, | 95,00% CI  of diff, | Below threshold? | Summary | Adjusted  P Value |
| --- | --- | --- | --- | --- | --- |
| Prolintane vs. 2-Methylprolintane | -0.5539 | -0.7118 to -0.3960 | Yes | **** | <0.0001 |
| Prolintane vs. 3-Methylprolintane | -0.2015 | -0.3594 to -0.04358 | Yes | ** | 0.0091 |
| Prolintane vs. 4-Methylprolintane | -0.06797 | -0.2259 to 0.08992 | No | ns | 0.757 |
| Prolintane vs. 2-Fluoroprolintane | -0.2108 | -0.3687 to -0.05287 | Yes | ** | 0.0063 |
| Prolintane vs. 3-Fluoroprolintane | 0.1468 | -0.01107 to 0.3047 | No | ns | 0.0762 |
| Prolintane vs. 4-Fluoroprolintane | -0.7709 | -0.9288 to -0.6130 | Yes | **** | <0.0001 |
| 2-Methylprolintane  vs. 3-Methylprolintane | 0.3524 | 0.1945 to 0.5103 | Yes | **** | <0.0001 |
| 2-Methylprolintane  vs. 4-Methylprolintane | 0.4859 | 0.3280 to 0.6438 | Yes | **** | <0.0001 |
| 2-Methylprolintane  vs. 2-Fluoroprolintane | 0.3431 | 0.1852 to 0.5010 | Yes | **** | <0.0001 |
| 2-Methylprolintane  vs. 3-Fluoroprolintane | 0.7007 | 0.5428 to 0.8586 | Yes | **** | <0.0001 |
| 2-Methylprolintane  vs. 4-Fluoroprolintane | -0.217 | -0.3749 to -0.05909 | Yes | ** | 0.0049 |
| 3-Methylprolintane vs. 4-Methylprolintane | 0.1335 | -0.02440 to 0.2914 | No | ns | 0.1245 |
| 3-Methylprolintane vs. 2-Fluoroprolintane | -0.009295 | -0.1672 to 0.1486 | No | ns | >0.9999 |
| 3-Methylprolintane vs. 3-Fluoroprolintane | 0.3483 | 0.1904 to 0.5062 | Yes | **** | <0.0001 |
| 3-Methylprolintane vs. 4-Fluoroprolintane | -0.5694 | -0.7273 to -0.4115 | Yes | **** | <0.0001 |
| 4-Methylprolintane vs. 2-Fluoroprolintane | -0.1428 | -0.3007 to 0.01510 | No | ns | 0.0886 |
| 4-Methylprolintane vs. 3-Fluoroprolintane | 0.2148 | 0.05690 to 0.3727 | Yes | ** | 0.0054 |
| 4-Methylprolintane vs. 4-Fluoroprolintane | -0.7029 | -0.8608 to -0.5450 | Yes | **** | <0.0001 |
| 2-Fluoroprolintane vs. 3-Fluoroprolintane | 0.3576 | 0.1997 to 0.5155 | Yes | **** | <0.0001 |
| 2-Fluoroprolintane vs. 4-Fluoroprolintane | -0.5601 | -0.7180 to -0.4022 | Yes | **** | <0.0001 |
| 3-Fluoroprolintane  vs. 4-Fluoroprolintane | -0.9177 | -1.076 to -0.7598 | Yes | **** | <0.0001 |

- 1. ***Batch Release Assays Statistics***

**Table S15**: Summary of two-way ANOVA of hSERT-mediated efflux data (batch release).

| ANOVA table | SS (Type III) | DF | MS | F (DFn, DFd) | P value |
| --- | --- | --- | --- | --- | --- |
| Interaction | 399.3 | 8 | 49.91 | F (8, 83) = 7.899 | P<0.0001 |
| Row Factor | 2868 | 8 | 358.5 | F (8, 83) = 56.73 | P<0.0001 |
| Column Factor | 1101 | 1 | 1101 | F (1, 83) = 174.3 | P<0.0001 |
| Residual | 524.5 | 83 | 6.319 |  |  |

**Table S16**: Summary of Sidak’s multiple comparisons test of hSERT-mediated efflux data (batch release).

| Šídák's multiple  comparisons test | Predicted (LS)  Mean diff, | 95,00% CI  of diff, | Below threshold? | Summary | Adjusted  P Value |
| --- | --- | --- | --- | --- | --- |
| No mon - Mon |  |  |  |  |  |
| Prolintane | -7.218 | -11.73 to -2.704 | Yes | *** | 0.0002 |
| 2-Me-Prolintane | -4.459 | -8.781 to -0.1374 | Yes | * | 0.0387 |
| 3-Me-Prolintane | -5.6 | -9.721 to -1.479 | Yes | ** | 0.002 |
| 4-Me-Prolintane | -4.314 | -7.882 to -0.7451 | Yes | ** | 0.0084 |
| 2-F-Prolintane | -6.392 | -10.51 to -2.271 | Yes | *** | 0.0003 |
| 3-F-Prolintane | -5.404 | -9.524 to -1.283 | Yes | ** | 0.0032 |
| 4-F-Prolintane | -7.206 | -11.33 to -3.085 | Yes | **** | <0.0001 |
| pCA | -18.87 | -23.91 to -13.82 | Yes | **** | <0.0001 |
| Paroxetine | -1.287 | -6.334 to 3.760 | No | ns | 0.9968 |

**Table S17**: Summary of two-way ANOVA of hDAT-mediated efflux data (batch release).

| ANOVA table | SS (Type III) | DF | MS | F (DFn, DFd) | P value |
| --- | --- | --- | --- | --- | --- |
| Interaction | 6.325 | 8 | 0.7906 | F (8, 69) = 1,897 | P=0.0744 |
| Row Factor | 170.8 | 8 | 21.34 | F (8, 69) = 51,21 | P<0.0001 |
| Column Factor | 6.231 | 1 | 6.231 | F (1, 69) = 14,95 | P=0.0002 |
| Residual | 28.76 | 69 | 0.4168 |  |  |

**Table S18**: Summary of Sidak’s multiple comparisons test of hDAT-mediated efflux data (batch release).

| Šídák's multiple  comparisons test | Predicted (LS)  Mean diff, | 95,00% CI  of diff, | Below threshold? | Summary | Adjusted  P Value |
| --- | --- | --- | --- | --- | --- |
| No mon - Mon |  |  |  |  |  |
| Prolintane | 0.1383 | -0.9258 to 1.202 | No | ns | >0.9999 |
| 2-Me-Prolintane | -0.04333 | -1.107 to 1.021 | No | ns | >0.9999 |
| 3-Me-Prolintane | -0.702 | -1.818 to 0.4141 | No | ns | 0.5135 |
| 4-Me-Prolintane | -0.8967 | -1.961 to 0.1674 | No | ns | 0.1573 |
| 2-F-Prolintane | -0.6425 | -1.946 to 0.6608 | No | ns | 0.8001 |
| 3-F-Prolintane | 0.0325 | -1.271 to 1.336 | No | ns | >0.9999 |
| 4-F-Prolintane | -0.2875 | -1.591 to 1.016 | No | ns | 0.9989 |
| S-(+)-amphetamine | -1.735 | -3.038 to -0.4317 | Yes | ** | 0.0028 |
| GBR12909 | -0.7725 | -2.076 to 0.5308 | No | ns | 0.5933 |

**Table S19**: Summary of two-way ANOVA of hNET-mediated efflux data (batch release).

| ANOVA table | SS (Type III) | DF | MS | F (DFn, DFd) | P value |
| --- | --- | --- | --- | --- | --- |
| Interaction | 82.73 | 8 | 10.34 | F (8, 119) = 5.517 | P<0.0001 |
| Row Factor | 1387 | 8 | 173.4 | F (8, 119) = 92.52 | P<0.0001 |
| Column Factor | 19.28 | 1 | 19.28 | F (1, 119) = 10.29 | P=0.0017 |
| Residual | 223 | 119 | 1.874 |  |  |

**Table S20**: Summary of Sidak’s multiple comparisons test of hNET-mediated efflux data (batch release).

| Šídák's multiple  comparisons test | Predicted (LS)  Mean diff, | 95,00% CI  of diff, | Below threshold? | Summary | Adjusted  P Value |
| --- | --- | --- | --- | --- | --- |
| No mon - Mon |  |  |  |  |  |
| Prolintane | -0.3446 | -2.273 to 1.583 | No | ns | 0.9998 |
| 2-Me-Prolintane | -0.3417 | -2.568 to 1.885 | No | ns | >0.9999 |
| 3-Me-Prolintane | -0.8634 | -2.791 to 1.065 | No | ns | 0.8797 |
| 4-Me-Prolintane | -0.3451 | -2.273 to 1.583 | No | ns | 0.9998 |
| 2-F-Prolintane | 0.3867 | -1.385 to 2.158 | No | ns | 0.9991 |
| 3-F-Prolintane | 0.3678 | -1.404 to 2.140 | No | ns | 0.9994 |
| 4-F-Prolintane | 0.3845 | -1.611 to 2.380 | No | ns | 0.9997 |
| S-(+)-amphetamine | -5.397 | -7.623 to -3.171 | Yes | **** | <0.0001 |
| GBR12909 | -0.7072 | -2.933 to 1.519 | No | ns | 0.985 |

- 1. ***Superfusion Release Assays Statistics***

**Table S21**: Summary of Mixed-effects model (REML) of hSERT efflux by prolintane ± Mon.

| Fixed effects (type III) | P value | P value  summary | Statistically sign.  (P < 0,05)? | F (DFn, DFd) | Geisser-Greenhouse's  epsilon |
| --- | --- | --- | --- | --- | --- |
| Time | <0.0001 | **** | Yes | F (1.195, 18.14) = 225.1 | 0.1086 |
| Column Factor | <0.0001 | **** | Yes | F (1, 16) = 68.87 |  |
| Time x Column Factor | <0.0001 | **** | Yes | F (1.195, 18.14) = 131.6 | 0.1086 |

**Table S22**: Summary of Sidak’s multiple comparisons test of hSERT efflux by prolintane ± Mon.

| Šídák's multiple  comparisons test | Mean diff, | 95,00% CI  of diff, | Below threshold? | Summary | Adjusted  P Value |
| --- | --- | --- | --- | --- | --- |
| Row 1 | 0.02682 | -0.2935 to 0.3471 | No | ns | >0.9999 |
| Row 2 | 0.1025 | -0.3017 to 0.5067 | No | ns | 0.9952 |
| Row 3 | 0.1806 | -0.6140 to 0.9751 | No | ns | 0.9968 |
| Row 4 | 0.1203 | -0.6375 to 0.8782 | No | ns | >0.9999 |
| Row 5 | 0.07151 | -0.5536 to 0.6967 | No | ns | >0.9999 |
| Row 6 | -0.1392 | -0.5580 to 0.2797 | No | ns | 0.9666 |
| Row 7 | -0.1744 | -0.5480 to 0.1991 | No | ns | 0.785 |
| Row 8 | -5.479 | -8.240 to -2.717 | Yes | *** | 0.0003 |
| Row 9 | -6.902 | -9.101 to -4.703 | Yes | **** | <0.0001 |
| Row 10 | -6.703 | -8.605 to -4.801 | Yes | **** | <0.0001 |
| Row 11 | -6.503 | -8.358 to -4.647 | Yes | **** | <0.0001 |
| Row 12 | -6.083 | -7.739 to -4.428 | Yes | **** | <0.0001 |

**Table S23**: Summary of Mixed-effects model (REML) of hSERT efflux by 2-Me-prolintane ± Mon.

| Fixed effects (type III) | P value | P value  summary | Statistically sign.  (P < 0,05)? | F (DFn, DFd) | Geisser-Greenhouse's  epsilon |
| --- | --- | --- | --- | --- | --- |
| Time | <0.0001 | **** | Yes | F (1.397, 19.44) = 340,5 | 0.1270 |
| Column Factor | <0.0001 | **** | Yes | F (1, 14) = 129.7 |  |
| Time x Column Factor | <0.0001 | **** | Yes | F (1.397, 19.44) = 172,1 | 0.1270 |

**Table S24**: Summary of Sidak’s multiple comparisons test of hSERT efflux by 2-Me-prolintane ± Mon.

| Šídák's multiple  comparisons test | Mean diff, | 95,00% CI  of diff, | Below threshold? | Summary | Adjusted  P Value |
| --- | --- | --- | --- | --- | --- |
| Row 1 | 0.1289 | -0.3031 to 0.5608 | No | ns | 0.9805 |
| Row 2 | 0.1185 | -0.3454 to 0.5825 | No | ns | 0.9946 |
| Row 3 | 0.1694 | -0.2689 to 0.6076 | No | ns | 0.8759 |
| Row 4 | 0.04832 | -0.1784 to 0.2751 | No | ns | 0.9994 |
| Row 5 | -0.1068 | -0.3456 to 0.1320 | No | ns | 0.8465 |
| Row 6 | -0.2079 | -0.4759 to 0.06008 | No | ns | 0.2068 |
| Row 7 | -0.2202 | -0.5676 to 0.1273 | No | ns | 0.451 |
| Row 8 | -5.467 | -7.928 to -3.006 | Yes | **** | <0.0001 |
| Row 9 | -7.92 | -9.840 to -6.000 | Yes | **** | <0.0001 |
| Row 10 | -7.744 | -9.505 to -5.982 | Yes | **** | <0.0001 |
| Row 11 | -7.355 | -8.919 to -5.792 | Yes | **** | <0.0001 |
| Row 12 | -7.467 | -8.990 to -5.944 | Yes | **** | <0.0001 |

**Table S25**: Summary of Mixed-effects model (REML) of hSERT efflux by 3-Me-prolintane ± Mon.

| Fixed effects (type III) | P value | P value  summary | Statistically sign.  (P < 0,05)? | F (DFn, DFd) | Geisser-Greenhouse's  epsilon |
| --- | --- | --- | --- | --- | --- |
| Time | <0.0001 | **** | Yes | F (1.267, 20.27) = 367.9 | 0.1152 |
| Column Factor | <0.0001 | **** | Yes | F (1, 16) = 121.3 |  |
| Time x Column Factor | <0.0001 | **** | Yes | F (1.267, 20.27) = 212.4 | 0.1152 |

**Table S26**: Summary of Sidak’s multiple comparisons test of hSERT efflux by 3-Me-prolintane ± Mon.

| Šídák's multiple  comparisons test | Mean diff, | 95,00% CI  of diff, | Below threshold? | Summary | Adjusted  P Value |
| --- | --- | --- | --- | --- | --- |
| Row 1 | 0.05456 | -0.2922 to 0.4013 | No | ns | >0.9999 |
| Row 2 | 0.01844 | -0.2447 to 0.2816 | No | ns | >0.9999 |
| Row 3 | 0.02833 | -0.2541 to 0.3107 | No | ns | >0.9999 |
| Row 4 | 0.07733 | -0.4010 to 0.5557 | No | ns | >0.9999 |
| Row 5 | -0.1496 | -0.4586 to 0.1595 | No | ns | 0.796 |
| Row 6 | -0.229 | -0.5297 to 0.07169 | No | ns | 0.2321 |
| Row 7 | -0.329 | -0.6271 to -0.03094 | Yes | * | 0.0247 |
| Row 8 | -4.546 | -6.149 to -2.942 | Yes | **** | <0.0001 |
| Row 9 | -6.038 | -7.751 to -4.324 | Yes | **** | <0.0001 |
| Row 10 | -6.018 | -7.681 to -4.355 | Yes | **** | <0.0001 |
| Row 11 | -5.904 | -7.510 to -4.299 | Yes | **** | <0.0001 |
| Row 12 | -5.776 | -7.352 to -4.201 | Yes | **** | <0.0001 |

**Table S27**: Summary of Mixed-effects model (REML) of hSERT efflux by 4-Me-prolintane ± Mon.

| Fixed effects (type III) | P value | P value  summary | Statistically sign.  (P < 0,05)? | F (DFn, DFd) | Geisser-Greenhouse's  epsilon |
| --- | --- | --- | --- | --- | --- |
| Time | <0.0001 | **** | Yes | F (1.278, 20.44) = 303.1 | 0.1162 |
| Column Factor | <0.0001 | **** | Yes | F (1, 16) = 85.95 |  |
| Time x Column Factor | <0.0001 | **** | Yes | F (1.278, 20.44) = 151.8 | 0.1162 |

**Table S28**: Summary of Sidak’s multiple comparisons test of hSERT efflux by 4-Me-prolintane ± Mon.

| Šídák's multiple  comparisons test | Mean diff, | 95,00% CI  of diff, | Below threshold? | Summary | Adjusted  P Value |
| --- | --- | --- | --- | --- | --- |
| Row 1 | -0,002444 | -0,3418 to 0,3369 | No | ns | >0,9999 |
| Row 2 | 0,005667 | -0,3009 to 0,3123 | No | ns | >0,9999 |
| Row 3 | -0,04278 | -0,3740 to 0,2884 | No | ns | >0,9999 |
| Row 4 | -0,1390 | -0,5171 to 0,2391 | No | ns | 0,9465 |
| Row 5 | -0,3006 | -0,7145 to 0,1133 | No | ns | 0,2597 |
| Row 6 | -0,3681 | -0,7698 to 0,03362 | No | ns | 0,0838 |
| Row 7 | -0,4242 | -0,7738 to -0,07467 | Yes | * | 0,0137 |
| Row 8 | -4,103 | -5,860 to -2,346 | Yes | *** | 0,0001 |
| Row 9 | -5,748 | -7,818 to -3,677 | Yes | **** | <0,0001 |
| Row 10 | -5,723 | -7,775 to -3,670 | Yes | **** | <0,0001 |
| Row 11 | -5,744 | -7,680 to -3,808 | Yes | **** | <0,0001 |
| Row 12 | -5,949 | -7,676 to -4,222 | Yes | **** | <0,0001 |

**Table S29**: Summary of Mixed-effects model (REML) of hSERT efflux by 2-F-prolintane ± Mon.

| Fixed effects (type III) | P value | P value  summary | Statistically sign.  (P < 0,05)? | F (DFn, DFd) | Geisser-Greenhouse's  epsilon |
| --- | --- | --- | --- | --- | --- |
| Time | <0.0001 | **** | Yes | F (1.706, 27.30) = 316.5 | 0.1551 |
| Column Factor | <0.0001 | **** | Yes | F (1, 16) = 288.3 |  |
| Time x Column Factor | <0.0001 | **** | Yes | F (1.706, 27.30) = 210.2 | 0.1551 |

**Table S30**: Summary of Sidak’s multiple comparisons test of hSERT efflux by 2-F-prolintane ± Mon.

| Šídák's multiple  comparisons test | Mean diff, | 95,00% CI  of diff, | Below threshold? | Summary | Adjusted  P Value |
| --- | --- | --- | --- | --- | --- |
| Row 1 | -0.05533 | -0.3045 to 0.1938 | No | ns | 0.9995 |
| Row 2 | -0.01811 | -0.2708 to 0.2345 | No | ns | >0.9999 |
| Row 3 | -0.031 | -0.2347 to 0.1727 | No | ns | >0.9999 |
| Row 4 | -0.05989 | -0.2646 to 0.1449 | No | ns | 0.9936 |
| Row 5 | -0.128 | -0.3842 to 0.1282 | No | ns | 0.7697 |
| Row 6 | -0.2267 | -0.4771 to 0.02375 | No | ns | 0.0948 |
| Row 7 | -0.3166 | -0.5536 to -0.07953 | Yes | ** | 0.0049 |
| Row 8 | -4.727 | -7.272 to -2.182 | Yes | *** | 0.001 |
| Row 9 | -7.079 | -8.503 to -5.655 | Yes | **** | <0.0001 |
| Row 10 | -7.103 | -8.426 to -5.779 | Yes | **** | <0.0001 |
| Row 11 | -6.897 | -8.117 to -5.677 | Yes | **** | <0.0001 |
| Row 12 | -6.561 | -7.615 to -5.508 | Yes | **** | <0.0001 |

**Table S31**: Summary of Mixed-effects model (REML) of hSERT efflux by 3-F-prolintane ± Mon.

| Fixed effects (type III) | P value | P value  summary | Statistically sign.  (P < 0,05)? | F (DFn, DFd) | Geisser-Greenhouse's  epsilon |
| --- | --- | --- | --- | --- | --- |
| Time | <0.0001 | **** | Yes | F (1.865, 29.67) = 430.3 | 0.1696 |
| Column Factor | <0.0001 | **** | Yes | F (1, 16) = 814.8 |  |
| Time x Column Factor | <0.0001 | **** | Yes | F (1.865, 29.67) = 276.3 | 0.1696 |

**Table S32**: Summary of Sidak’s multiple comparisons test of hSERT efflux by 3-F-prolintane ± Mon.

| Šídák's multiple  comparisons test | Mean diff, | 95,00% CI  of diff, | Below threshold? | Summary | Adjusted  P Value |
| --- | --- | --- | --- | --- | --- |
| Row 1 | 0,003889 | -0,2860 to 0,2938 | No | ns | >0,9999 |
| Row 2 | -0,003000 | -0,2809 to 0,2749 | No | ns | >0,9999 |
| Row 3 | 0,02067 | -0,2532 to 0,2945 | No | ns | >0,9999 |
| Row 4 | -0,01133 | -0,2565 to 0,2338 | No | ns | >0,9999 |
| Row 5 | -0,1369 | -0,3916 to 0,1178 | No | ns | 0,6632 |
| Row 6 | -0,2296 | -0,4686 to 0,009468 | No | ns | 0,0649 |
| Row 7 | -0,2910 | -0,5458 to -0,03619 | Yes | * | 0,0197 |
| Row 8 | -4,827 | -6,988 to -2,666 | Yes | *** | 0,0002 |
| Row 9 | -7,510 | -8,391 to -6,629 | Yes | **** | <0,0001 |
| Row 10 | -7,391 | -8,365 to -6,416 | Yes | **** | <0,0001 |
| Row 11 | -7,140 | -8,101 to -6,179 | Yes | **** | <0,0001 |
| Row 12 | -7,011 | -8,172 to -5,850 | Yes | **** | <0,0001 |

**Table S33**: Summary of Mixed-effects model (REML) of hSERT efflux by 4-F-prolintane ± Mon.

| Fixed effects (type III) | P value | P value  summary | Statistically sign.  (P < 0,05)? | F (DFn, DFd) | Geisser-Greenhouse's  epsilon |
| --- | --- | --- | --- | --- | --- |
| Time | <0.0001 | **** | Yes | F (1.485, 23.76) = 442.5 | 0.135 |
| Column Factor | <0.0001 | **** | Yes | F (1, 16) = 1540 |  |
| Time x Column Factor | <0.0001 | **** | Yes | F (1.485, 23.76) = 288.3 | 0.135 |

**Table S34**: Summary of Sidak’s multiple comparisons test of hSERT efflux by 4-F-prolintane ± Mon.

| Šídák's multiple  comparisons test | Mean diff, | 95,00% CI  of diff, | Below threshold? | Summary | Adjusted  P Value |
| --- | --- | --- | --- | --- | --- |
| Row 1 | -0,0007778 | -0,2292 to 0,2276 | No | ns | >0,9999 |
| Row 2 | 0,02200 | -0,2265 to 0,2705 | No | ns | >0,9999 |
| Row 3 | 0,004889 | -0,2201 to 0,2299 | No | ns | >0,9999 |
| Row 4 | -0,08044 | -0,2641 to 0,1032 | No | ns | 0,8785 |
| Row 5 | -0,1983 | -0,3773 to -0,01941 | Yes | * | 0,0237 |
| Row 6 | -0,2323 | -0,4867 to 0,02199 | No | ns | 0,0853 |
| Row 7 | -0,3017 | -0,4815 to -0,1218 | Yes | *** | 0,0007 |
| Row 8 | -8,349 | -10,25 to -6,449 | Yes | **** | <0,0001 |
| Row 9 | -9,598 | -10,57 to -8,625 | Yes | **** | <0,0001 |
| Row 10 | -8,382 | -9,225 to -7,539 | Yes | **** | <0,0001 |
| Row 11 | -7,454 | -8,837 to -6,071 | Yes | **** | <0,0001 |
| Row 12 | -6,666 | -8,369 to -4,962 | Yes | **** | <0,0001 |

**Table S35**: Summary of Mixed-effects model (REML) of hSERT efflux by paroxetine ± Mon.

| Fixed effects (type III) | P value | P value  summary | Statistically sign.  (P < 0,05)? | F (DFn, DFd) | Geisser-Greenhouse's  epsilon |
| --- | --- | --- | --- | --- | --- |
| Time | <0.0001 | **** | Yes | F (1.190, 18.82) = 22.88 | 0.1082 |
| Column Factor | 0.0229 | * | Yes | F (1, 16) = 6.330 |  |
| Time x Column Factor | 0.0761 | ns | No | F (1.190, 18.82) = 3.377 | 0.1082 |

**Table S36**: Summary of Sidak’s multiple comparisons test of hSERT efflux by paroxetine ± Mon.

| Šídák's multiple  comparisons test | Mean diff, | 95,00% CI  of diff, | Below threshold? | Summary | Adjusted  P Value |
| --- | --- | --- | --- | --- | --- |
| Row 1 | 0.02389 | -0.3164 to 0.3642 | No | ns | >0.9999 |
| Row 2 | 0.01911 | -0.3499 to 0.3881 | No | ns | >0.9999 |
| Row 3 | -0.033 | -0.3988 to 0.3328 | No | ns | >0.9999 |
| Row 4 | -0.09222 | -0.4708 to 0.2864 | No | ns | 0.9988 |
| Row 5 | -0.292 | -0.6421 to 0.05813 | No | ns | 0.1503 |
| Row 6 | -0.4089 | -0.7269 to -0.09091 | Yes | ** | 0.007 |
| Row 7 | -0.4921 | -0.9074 to -0.07685 | Yes | * | 0.0143 |
| Row 8 | -0.6678 | -1.234 to -0.1014 | Yes | * | 0.0155 |
| Row 9 | -0.8309 | -1.970 to 0.3080 | No | ns | 0.2739 |
| Row 10 | -0.8986 | -2.341 to 0.5442 | No | ns | 0.4806 |
| Row 11 | -0.7665 | -2.334 to 0.8015 | No | ns | 0.7686 |
| Row 12 | -0.7707 | -2.488 to 0.9469 | No | ns | 0.8501 |

**Table S37**: Summary of Mixed-effects model (REML) of rSERT efflux by prolintane ± Mon.

| Fixed effects (type III) | P value | P value  summary | Statistically sign.  (P < 0,05)? | F (DFn, DFd) | Geisser-Greenhouse's  epsilon |
| --- | --- | --- | --- | --- | --- |
| Time | <0.0001 | **** | Yes | F (1.799, 14.39) = 42.85 | 0.1635 |
| Column Factor | 0.0003 | *** | Yes | F (1.000, 8.000) = 37.30 | 1 |
| Time x Column Factor | 0.0002 | *** | Yes | F (1.779, 13.90) = 17.87 | 0.1617 |

**Table S38**: Summary of Sidak’s multiple comparisons test of rSERT efflux by prolintane ± Mon.

| Šídák's multiple  comparisons test | Mean diff, | 95,00% CI  of diff, | Below threshold? | Summary | Adjusted  P Value |
| --- | --- | --- | --- | --- | --- |
| Row 1 | -0.3272 | -1.122 to 0.4680 | No | ns | 0.8434 |
| Row 2 | -0.2896 | -0.9753 to 0.3962 | No | ns | 0.8229 |
| Row 3 | -0.1547 | -0.9678 to 0.6584 | No | ns | 0.9996 |
| Row 4 | -0.119 | -0.5016 to 0.2636 | No | ns | 0.9706 |
| Row 5 | -0.6706 | -1.251 to -0.09043 | Yes | * | 0.022 |
| Row 6 | -0.9234 | -1.481 to -0.3657 | Yes | ** | 0.0022 |
| Row 7 | -1.427 | -2.524 to -0.3302 | Yes | * | 0.0107 |
| Row 8 | -2.621 | -4.637 to -0.6046 | Yes | * | 0.012 |
| Row 9 | -2.612 | -4.443 to -0.7813 | Yes | ** | 0.007 |
| Row 10 | -2.362 | -4.050 to -0.6737 | Yes | ** | 0.0067 |
| Row 11 | -2.416 | -3.972 to -0.8593 | Yes | ** | 0.0034 |
| Row 12 | -2.279 | -4.139 to -0.4196 | Yes | * | 0.0154 |

**Table S39**: Summary of Mixed-effects model (REML) of rSERT efflux by paroxetine ± Mon.

| Fixed effects (type III) | P value | P value  summary | Statistically sign.  (P < 0,05)? | F (DFn, DFd) | Geisser-Greenhouse's  epsilon |
| --- | --- | --- | --- | --- | --- |
| Time | <0.0001 | **** | Yes | F (2.634, 41.90) = 36.92 | 0.2394 |
| Column Factor | 0.001 | ** | Yes | F (1, 16) = 16.10 |  |
| Time x Column Factor | 0.0001 | *** | Yes | F (2.634, 41.90) = 9.663 | 0.2394 |

**Table S40**: Summary of Sidak’s multiple comparisons test of rSERT efflux by paroxetine ± Mon.

| Šídák's multiple  comparisons test | Mean diff, | 95,00% CI  of diff, | Below threshold? | Summary | Adjusted  P Value |
| --- | --- | --- | --- | --- | --- |
| Row 1 | 0.1442 | -0.9940 to 1.282 | No | ns | >0.9999 |
| Row 2 | -0.02622 | -1.164 to 1.112 | No | ns | >0.9999 |
| Row 3 | -0.1814 | -1.135 to 0.7721 | No | ns | 0.9999 |
| Row 4 | -0.04233 | -0.7686 to 0.6840 | No | ns | >0.9999 |
| Row 5 | -0.6121 | -1.395 to 0.1711 | No | ns | 0.2086 |
| Row 6 | -1.044 | -1.784 to -0.3037 | Yes | ** | 0.0029 |
| Row 7 | -1.227 | -2.020 to -0.4345 | Yes | ** | 0.0013 |
| Row 8 | -1.361 | -2.188 to -0.5341 | Yes | ** | 0.0011 |
| Row 9 | -2.004 | -3.251 to -0.7575 | Yes | ** | 0.001 |
| Row 10 | -1.579 | -3.180 to 0.02230 | No | ns | 0.0549 |
| Row 11 | -1.619 | -3.004 to -0.2351 | Yes | * | 0.0155 |
| Row 12 | -1.624 | -3.096 to -0.1527 | Yes | * | 0.025 |

**Table S41**: Summary of Mixed-effects model (REML) of hSERT efflux by pCA ± Mon.

| Fixed effects (type III) | P value | P value  summary | Statistically sign.  (P < 0,05)? | F (DFn, DFd) | Geisser-Greenhouse's  epsilon |
| --- | --- | --- | --- | --- | --- |
| Time | <0.0001 | **** | Yes | F (1.913, 30.43) = 655.0 | 0.1739 |
| Column Factor | <0.0001 | **** | Yes | F (1, 16) = 102.5 |  |
| Time x Column Factor | <0.0001 | **** | Yes | F (1.913, 30.43) = 169.2 | 0.1739 |

**Table S42**: Summary of Sidak’s multiple comparisons test of hSERT efflux by pCA ± Mon.

| Šídák's multiple  comparisons test | Mean diff, | 95,00% CI  of diff, | Below threshold? | Summary | Adjusted  P Value |
| --- | --- | --- | --- | --- | --- |
| Row 1 | 0.041 | -0.2152 to 0.2972 | No | ns | >0.9999 |
| Row 2 | -0.01744 | -0.2249 to 0.1900 | No | ns | >0.9999 |
| Row 3 | -0.07044 | -0.3305 to 0.1896 | No | ns | 0.9946 |
| Row 4 | -0.2019 | -0.5204 to 0.1166 | No | ns | 0.4287 |
| Row 5 | -0.4027 | -0.7454 to -0.05993 | Yes | * | 0.0166 |
| Row 6 | -0.4678 | -0.7621 to -0.1735 | Yes | ** | 0.0013 |
| Row 7 | -0.5487 | -0.8387 to -0.2586 | Yes | *** | 0.0002 |
| Row 8 | -39.17 | -47.26 to -31.08 | Yes | **** | <0.0001 |
| Row 9 | -13.2 | -18.36 to -8.029 | Yes | **** | <0.0001 |
| Row 10 | -2.691 | -6.918 to 1.535 | No | ns | 0.4292 |
| Row 11 | 0.7369 | -2.737 to 4.211 | No | ns | 0.9995 |
| Row 12 | 1.522 | -2.167 to 5.210 | No | ns | 0.8918 |

**Table S43**: Summary of Mixed-effects model (REML) of rSERT efflux by pCA ± Mon.

| Fixed effects (type III) | P value | P value  summary | Statistically sign.  (P < 0,05)? | F (DFn, DFd) | Geisser-Greenhouse's  epsilon |
| --- | --- | --- | --- | --- | --- |
| Time | <0.0001 | **** | Yes | F (2.307, 36.50) = 171.5 | 0.2098 |
| Column Factor | <0.0001 | **** | Yes | F (1, 16) = 57.11 |  |
| Time x Column Factor | <0.0001 | **** | Yes | F (2.307, 36.50) = 63.15 | 0.2098 |

**Table S44**: Summary of Sidak’s multiple comparisons test of rSERT efflux by pCA ± Mon.

| Šídák's multiple  comparisons test | Mean diff, | 95,00% CI  of diff, | Below threshold? | Summary | Adjusted  P Value |
| --- | --- | --- | --- | --- | --- |
| Row 1 | 0.1306 | -0.5345 to 0.7956 | No | ns | 0.9999 |
| Row 2 | 0.1761 | -0.4115 to 0.7637 | No | ns | 0.9923 |
| Row 3 | 0.2854 | -0.4707 to 1.042 | No | ns | 0.9365 |
| Row 4 | 0.1377 | -0.8094 to 1.085 | No | ns | >0.9999 |
| Row 5 | -0.4218 | -0.9885 to 0.1449 | No | ns | 0.2491 |
| Row 6 | -0.8916 | -1.507 to -0.2766 | Yes | ** | 0.0036 |
| Row 7 | -1.153 | -1.748 to -0.5577 | Yes | *** | 0.0001 |
| Row 8 | -7.649 | -9.300 to -5.997 | Yes | **** | <0.0001 |
| Row 9 | -4.816 | -6.653 to -2.979 | Yes | **** | <0.0001 |
| Row 10 | -3.007 | -4.970 to -1.045 | Yes | ** | 0.0014 |
| Row 11 | -2.599 | -4.453 to -0.7452 | Yes | ** | 0.0034 |
| Row 12 | -2.043 | -3.787 to -0.2988 | Yes | * | 0.0157 |

- 1. ***Electrophysiology Descriptive Statistics***

**Table S45:** Descriptive statistics of *normalized (10 µM 5-HT) currents elicted through hSERT by drug application.*

|  | Prolintane | 2-Me-Prolintane | 3-Me-Prolintane | 4-Me-Prolintane | 2-F-Prolintane | 3-F-Prolintane | 4-F-Prolintane |
| --- | --- | --- | --- | --- | --- | --- | --- |
| Number of values | 6 | 5 | 5 | 4 | 5 | 5 | 6 |
| Minimum | 0.17 | 0.22 | 0.2 | 0.13 | 0.22 | 0.2 | 0.27 |
| Maximum | 0.38 | 0.44 | 0.47 | 0.3 | 0.36 | 0.39 | 0.38 |
| Range | 0.21 | 0.22 | 0.27 | 0.17 | 0.14 | 0.19 | 0.11 |
| Mean | 0.2567 | 0.386 | 0.354 | 0.21 | 0.28 | 0.306 | 0.3167 |
| Std.  Deviation | 0.07685 | 0.09343 | 0.1062 | 0.08287 | 0.05831 | 0.08264 | 0.05125 |
| Std. Error of Mean | 0.03138 | 0.04179 | 0.0475 | 0.04143 | 0.02608 | 0.03696 | 0.02092 |

- 1. ***Electrophysiology Additional Control Experiments***


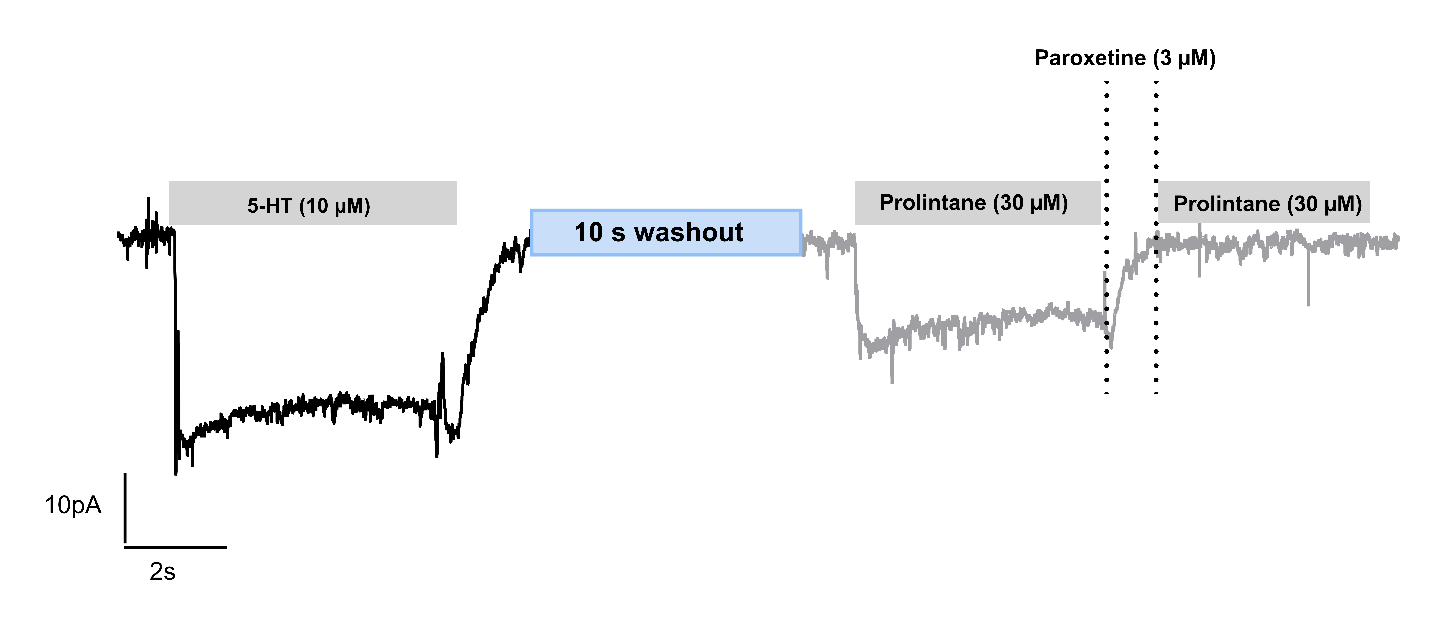


**Figure S2:** Representative current trace showing the effect of serotonin (5-HT, 10 µM) application on current amplitude, followed by a 10-second washout period. After washout, the cell was treated with prolintane (30 µM), which elicited an inward current. Subsequent application of the selective serotonin reuptake inhibitor paroxetine (3 µM), indicated by the black dotted lines, caused a complete reduction in the prolintane-induced current, suggesting that paroxetine inhibits the effect of prolintane on serotonin transporter-mediated current.


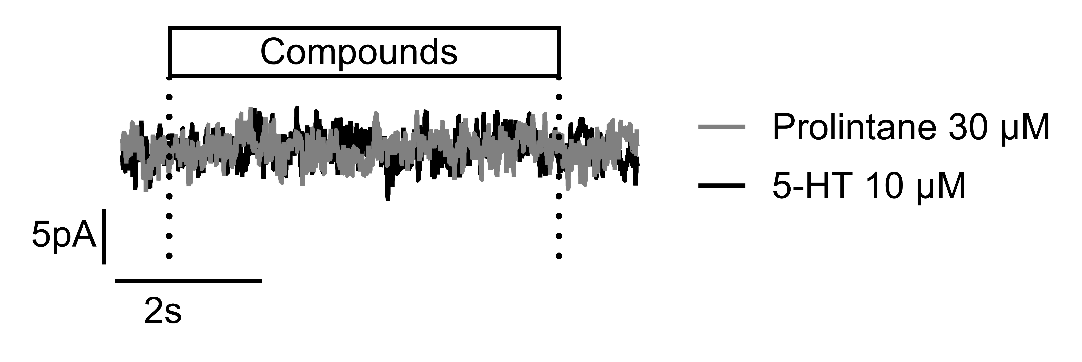


**Figure S3:** Prolintane and 5-HT do not induce currents in empty HEK293 cells. Representative current traces during application of prolintane (30 µM, gray trace) and 5-HT (10 µM, black trace). Application of either compound, indicated by the horizontal bar and by dotted lines, did not evoke an inward current, demonstrating that the observed effects in transfected cells are transporter-dependent.
